# Supplementary material for: Self-harm among unaccompanied asylum seekers and refugee minors: protocol for a global systematic review of prevalence, methods and characteristics
Source: BMJ Open. 2023 Jun 6;13(6):e069237. doi: 10.1136/bmjopen-2022-069237 (PMC10255047; doi:10.1136/bmjopen-2022-069237)
Supplement: Supplementary data [file bmjopen-2022-069237supp001.pdf]

Supplementary appendix 1

Overview of search strategies used for each database

MEDLINE (Ovid) search strategy filtered by date from database inception to 10 February 2023

1. (non-suicidal self-harm or self-injur* or deliberate self-harm or self-poison* or self-mutilat*).af.
2. (migration or immigration or unaccompanied asylum seeker or unaccompanied refugee minor or child or adolescent or teenager).af.
3. 1 and 2

PsycINFO (Ovid) search strategy filtered by date from database inception to 10 February 2023

1. (non-suicidal self-harm or self-injur* or deliberate self-harm or self-poison* or self-mutilat*).af.
2. (migration or immigration or unaccompanied asylum seeker or unaccompanied refugee minor or child or adolescent or teenager).af.
3. 1 and 2

PubMed search strategy filtered by date from database inception to 10 February 2023

(((non-suicidal self-harm or self-injur* or deliberate self-harm or self-poison* or self-mutilat*)) OR ((migration or immigration or unaccompanied asylum seeker or unaccompanied refugee minor or child or adolescent or teenager).af.)) AND (non-suicidal self-harm or self-injur* or deliberate self-harm or self-poison* or self-mutilat* AND migration or immigration or unaccompanied asylum seeker or unaccompanied refugee minor or child or adolescent or teenager)

Scopus search strategy

{non-suicidal self-harm} or {self-injur*} or {deliberate self-harm} or {self-poison*} or {self-mutilat*} OR {migration} or {immigration} or {unaccompanied asylum seeker} or {unaccompanied refugee minor} or child or adolescent or teenager).af.)) AND {non-suicidal self-harm} or {self-injur*} or {deliberate self-harm} or {self-poison*} or {self-mutilat*} AND {migration} or {immigration} or {unaccompanied asylum seeker} or {unaccompanied refugee minor} or child or adolescent or teenager)
