# Supplementary material for: Self-harm among unaccompanied asylum seekers and refugee minors: protocol for a global systematic review of prevalence, methods and characteristics
Source: BMJ Open. 2023 Jun 6;13(6):e069237. doi: 10.1136/bmjopen-2022-069237 (PMC10255047; doi:10.1136/bmjopen-2022-069237)
Supplement: Author's manuscript [file bmjopen-2022-069237.draft_revisions.pdf]

BMJ Open is committed to open peer review. As part of this commitment we make the peer review history of every article we publish publicly available.

When an article is published we post the peer reviewers' comments and the authors' responses online. We also post the versions of the paper that were used during peer review. These are the versions that the peer review comments apply to.

The versions of the paper that follow are the versions that were submitted during the peer review process. They are not the versions of record or the final published versions. They should not be cited or distributed as the published version of this manuscript.

BMJ Open is an open access journal and the full, final, typeset and author-corrected version of record of the manuscript is available on our site with no access controls, subscription charges or pay-per-view fees (http://bmjopen.bmj.com).

If you have any questions on BMJ Open's open peer review process please email
info.bmjopen@bmj.com

BMJ Open

Self-harm among unaccompanied asylum seekers and refugee minors: protocol for a global systematic review of prevalence, methods, and characteristics

Journal:	*BMJ Open*
Manuscript ID	bmjopen-2022-069237
Article Type:	Protocol
Date Submitted by the Author:	14-Oct-2022
Complete List of Authors:	Hedrick, Kyli; Uppsala Universitet, Department of Public Health and Caring Sciences; The University of Melbourne - Parkville Campus, Centre for Health Equity, Melbourne School of Population and Global Health<br>Borschmann, Rohan; The University of Melbourne, Centre for Health Equity, Melbourne School of Population and Global Health; Oxford University, Department of Psychiatry, Warneford Hospital
Keywords:	MENTAL HEALTH, PUBLIC HEALTH, Suicide & self-harm < PSYCHIATRY, Child & adolescent psychiatry < PSYCHIATRY

SCHOLARONE™ Manuscripts

Self-harm among unaccompanied asylum seekers and refugee minors: protocol for a global systematic review of prevalence, methods, and characteristics

Kyli Hedrick[1,2], Rohan Borschmann[2,3,4,5]

[1]Child Health and Parenting (CHAP), Department of Public Health and Caring Sciences, Uppsala University, BMC, Husargatan 3, 753 27, Uppsala, Sweden.

[2]Justice Health Unit, Centre for Health Equity; Melbourne School of Population and Global Health, The University of Melbourne, Melbourne, Victoria, 3010, Australia

[3]Department of Psychiatry; Warneford Hospital, University of Oxford, Oxford OX3 7JX, UK

[4]Centre for Adolescent Health; Murdoch Children's Research Institute, Melbourne, Victoria, 3052, Australia

[5]Melbourne School of Psychological Sciences; University of Melbourne, Parkville, 3010, Australia

Corresponding author: Dr. Kyli Hedrick

Email: kyli.hedrick@pubcare.uu.se

For peer review only

Abstract

***Introduction*:** Asylum seekers and refugees are at an elevated risk of self-harm, with younger age and traumatic experiences found to further increase such risk. Despite this, evidence regarding self-harm among unaccompanied asylum seekers and refugee minors has not yet been synthesised. As self-harm among minors is a risk factor for a range of adverse clinical and social outcomes, including suicide, such information may help to inform evidence-based prevention strategies among these vulnerable populations. This systematic review will synthesise findings from the literature regarding the prevalence, methods, and characteristics of self-harm, including risk and protective factors, among unaccompanied asylum seekers and refugee minors internationally.

***Methods and analysis*:** We will search key electronic health, psychology, and medical databases (PsycINFO, Scopus, PubMed, and Ovid MEDLINE) for relevant studies published in English from database inception. Our primary outcome of interest is self-harm among unaccompanied asylum seekers and/or refugee minors. We will include all types of study design (except single case studies) that examine the prevalence of self-harm in unaccompanied asylum seekers and/or refugee minors. We will restrict study eligibility by age of participants, so that only studies reporting on participants aged <18 years will be eligible for inclusion. The Methodological Standard for Epidemiological Research (MASTER) scale will be used to assess the quality of included studies. If there are sufficient studies, and homogeneity between them, we will conduct meta-analyses to calculate pooled estimates of self-harm rates, as well as comparisons between sub-groups of relevance. If the studies do not report sufficient data, or there is substantial heterogeneity, we will provide a narrative synthesis of the findings.

For peer review only - http://bmjopen.bmj.com/site/about/guidelines.xhtml

Ethics and dissemination: This review is exempt from ethics approval as it will synthesise findings from published studies that have already obtained ethics approval. Our findings will be disseminated through a peer-reviewed journal article and conference presentations.

PROSPERO registration number CRD42021292709.

Strengths and limitations of this study

- This systematic review uses a comprehensive search strategy including four key academic databases and a grey literature search.
- A strength of this study is that only studies involving unaccompanied asylum seekers and refugee minors and other immigrant populations, and that distinguish between these populations (i.e., consider these populations to be heterogenous), will be included.
- Studies that examine self-harm and suicide but do not distinguish between these acts will be excluded as these two outcomes are qualitatively and motivationally distinct from one another.
- A small number of primary studies, and heterogeneity between study populations and/or study design, may preclude meta-analysis or direct comparison between studies.

For peer review only

For peer review only - http://bmjopen.bmj.com/site/about/guidelines.xhtml

Introduction

Globally, the number of people forcibly displaced because of war, conflict, and political unrest has reached record highs. According to the United Nations High Commissioner for Refugees (UNHCR),[1] as at the end of May 2022, there are now over 100 million forcibly displaced people around the world. Children and young people are markedly over-represented in this humanitarian crisis, accounting for an estimated 41% of those displaced, though they make up 30% of the world's population.[2] Of the 24,100 children who arrived in Greece, Italy, Bulgaria, Spain, Cyprus, and Malta in the 12-month period to December 2021, 17,200 (71%) were unaccompanied and separated.[3] Unaccompanied asylum seeker and refugee minors are defined by the United Nations Convention on the Rights of the Child[4] as children or adolescents younger than 18 years who have been separated from both parents and relatives and are not being cared for by an adult, who, by law or custom, is responsible for doing so. First-time asylum applications from such minors in the EU Member States – for which data are available – increased by 10% between May and June 2022, and 63% between June 2021 and June 2022.[5] Whilst most unaccompanied minors are males aged between 14-17 years, numbers of female and younger populations are increasing.[6] In 2022, the main countries of origin for unaccompanied minors in European Union (EU) Member States are Afghanistan, Syria, Somalia, and Pakistan.[5] In the 2021 financial year, the United States (US) reported receiving an all-time record of unaccompanied minors from Central America at or near the US-Mexico border, including 145,000 minors.[7]

Many individuals who have been forcibly displaced have been subjected to a range of potentially traumatic events in the pre-, peri-, and post-migration phases.[8] These include witnessing the death of a family member or loved one, war, dangerous flight, poor humanitarian conditions, and periods of detention,[8 9] followed by the challenges associated with managing the uncertainty of the asylum process, and the resettlement process.[10] Young

For peer review only - http://bmjopen.bmj.com/site/about/guidelines.xhtml

people who have been forcibly displaced must navigate such adverse experiences during a challenging developmental period, which may further compound the detrimental impact of these events on their mental health.[11] For young people who are forcibly displaced without parents or caregivers, they must not only navigate several potentially traumatic experiences during a difficult time developmentally, but they must do so without the support and guidance of a parent or caregiver, and whilst managing the loss associated with separation from parents and/or family.[12] Upon arrival in host countries, unaccompanied minors may also be confronted with persistent questioning by authorities about their age, familial relationships, and reason(s) for leaving their home country.[13] In addition, they may be subjected to invasive and imprecise age assessments such as carpal, collarbone, and/or dental x-rays to establish the accuracy of their self-reported age.[14 15] The particular vulnerabilities and risks faced by unaccompanied asylum seekers and refugee minors have been increasingly acknowledged, paving the way for their entitlement to international protection,[4] as well as programs promoting their physical, mental, and educational development.[4 16]

Research has documented the numerous adverse mental health consequences for unaccompanied asylum seekers and refugee minors across several settings and locations.[17] For example, a 2019 global systematic review of the mental health of unaccompanied refugee minors by von Wethern and colleagues[17] found that despite varying estimates of psychological difficulties, rates of PTSD (17-85%), depression (13-76%), and anxiety (11-85%) were significantly higher in unaccompanied refugee minors than those found in the general western population. Evidence compiled by von Werthern et al.[17] also found that adolescence and being female were indicators of increased risk of psychiatric disorders. In addition, the authors' review highlighted that there was significantly more exposure to traumatic events such as physical violence, torture, imprisonment, and killings among unaccompanied minors, compared with exposure in the general population.[18-21]

For peer review only - http://bmjopen.bmj.com/site/about/guidelines.xhtml

Whilst earlier systematic reviews have largely focused on the mental health and wellbeing of unaccompanied minors,[17 22-24] or the mental health of refugee minors,[25] to our knowledge, no reviews have examined self-harm in unaccompanied asylum seeker and refugee minors globally. One broader review that focused on self-harming behaviours among immigrants in Europe by Garguilo and colleagues[26] did report – via a synthesis of three pertinent studies - that unaccompanied minors were an ‘at-risk’ group for self-harm in the European context. However, as unaccompanied minors were not explicitly included in Garguilo and colleagues’[26] search strategy, and the setting of their review was European, rather than global, it is likely that other salient studies may have been excluded. Asylum seekers and refugees are at increased risk of self-harm,[27] and this risk is further elevated in younger asylum seekers and refugees and those with experiences of trauma.[27 28] As such, a comprehensive global review synthesising the evidence relating to self-harm among unaccompanied asylum seekers and refugee minors could inform evidence-based prevention and management strategies in these vulnerable populations.

A Swedish study by Ramel and colleagues[29] was included in both the von Werthern[17] and Garguilo[26] reviews. In this study, the authors examined the psychiatric care of unaccompanied asylum seekers and reported that a higher proportion of unaccompanied refugee minors (76%) than accompanied minors (56%) exhibited self-harm or suicidal behaviour on admission to hospital. The study also noted that 86% of unaccompanied minors were hospitalised due to the stress associated with the asylum process.[29] A 2022 systematic review of the mental health of unaccompanied minors in Europe by Daniel-Calveras and colleagues[30] also included one study examining data from unaccompanied minors presenting to health care providers in the United Kingdom (UK), reporting that 8% had engaged in self-harm or suicidal behaviour.[31] Neither the reviews by von Werthern,[17] Garguilo,[26] or Daniel-Calveras and colleagues[30] (or the original authors of the two studies highlighted above[29 31]),

For peer review only - http://bmjopen.bmj.com/site/about/guidelines.xhtml

however, separated out or distinguished episodes of self-harm from suicidal behaviour when presenting their findings, though Garguilo and colleagues[26] did state in their discussion that self-harm should be viewed as a distinct clinical entity. As self-harm and suicidal behaviour are motivationally and qualitatively distinct from one another,[32] such a distinction is critical to inform the provision of appropriate physical and mental health care. Furthermore, as no terms relating to self-harm were included in von Werthern[17] or Daniel-Calveras's[30] search strategies, and those included in Garguilo et al.'s[26] search strategy were not exhaustive, additional studies focusing on self-harm (as distinct from suicidal behaviour) may not have been identified in these reviews.

We aim to synthesise the evidence regarding the prevalence, methods, and characteristics of self-harm, including risk and protective factors, among unaccompanied asylum seekers and refugee minors internationally.

Methods and analysis

This protocol is reported in accordance with the Preferred Reporting Items for Systematic Reviews and Meta-Analysis Protocols (PRISMA-P).[33]

Eligibility criteria

Participants

The UNHCR defines a refugee as a person who: 1) has a well-founded fear of being persecuted for reasons relating to race, religion, nationality, membership of a particular social group, or political opinion; 2) is outside the country of (their) nationality; and 3) is unable, or owing to such fear, is unwilling, to avail (themselves) of the protection of that country (p.14).[34] An asylum seeker is defined as an individual who is seeking refugee 'protection, but whose claim for refugee status has not yet been determined'.[35]

For peer review only - http://bmjopen.bmj.com/site/about/guidelines.xhtml

We will include studies examining self-harm among unaccompanied asylum seekers and refugee minors – defined as a person aged <18 years who has been separated from both parents and relatives and is not being cared for by an adult, who, by law or custom, is responsible for doing so[4] – in any country. Studies reporting on unaccompanied asylum seekers and/or refugee minors and other immigrant populations, but that do not distinguish between these populations, will be excluded. Studies reporting on suicide or suicide attempts only will be excluded. Where studies refer to suicide and self-harm separately, only findings regarding self-harm will be included.

Outcome measure

Our primary outcome measure will be self-harm. For the purposes of this review, self-harm is defined as any form of self-injury (or self-poisoning) irrespective of suicidal intent or motivation.[36] Self-harm may be measured by self-report, clinical interview, or administrative data (e.g., emergency department [ED], hospital, or incident reports). An additional outcome measure will be method(s) used to self-harm. Methods of self-harm may also be measured by self-report, clinical interview, or administrative data, including the World Health Organization's International Classification of Diseases Tenth Revision (ICD-10) codes.[37]

Study design

We will include published cohort studies of unaccompanied asylum seekers and refugee minors which report on the prevalence of self-harm. We will exclude single case studies, dissertations, conference abstracts, letters, book chapters, editorials, and study registrations. We will not include previous systematic reviews, as not all included studies may not meet our inclusion criteria. We will, however, identify any peer-reviewed studies related to any dissertations, conference abstracts, study registrations, and studies assessed for previous

For peer review only

For peer review only - http://bmjopen.bmj.com/site/about/guidelines.xhtml

reviews that meet our inclusion criteria but were not identified via our search strategy. Only studies published in English will be included.

Information sources and search strategy

We will search key health and medical databases (PsycINFO, Scopus, PubMed, and OVID MEDLINE) for relevant literature from database inception until the date of our final search. Variations of the following combinations of search terms will be used: self-harm OR non-suicidal self-harm OR non-suicidal self-injury (NSSI) OR self-harming behaviour OR self-injury OR self-injurious behaviour OR deliberate self-harm (DSH) OR DSH OR self-mutilation OR self-wounding OR self-poisoning OR self-inflicted violence OR parasuicide AND unaccompanied asylum seeker OR unaccompanied refugee minor OR child OR adolescent OR minor OR youth OR teenager OR forced migration OR immigration OR migration. The electronic database searches will be supplemented by reviewing the reference lists of eligible articles, as well as searching the websites of relevant non-governmental organisations who have worked with unaccompanied asylum seekers and refugee minors (such as Médecins sans Frontières and Save the Children), for pertinent grey literature.

Study selection

All studies identified through the database search will be downloaded to Endnote[38] and duplicates removed. The remaining studies will be imported into Covidence[39] for screening. All titles and abstracts will be screened for inclusion by the primary author (KH), with 20% screened by the second author (RB). After 20% of the papers identified in the search strategy have been double screened, we will reassess our eligibility criteria to ensure that they are relevant to the studies that are identified. The reassessment process will involve a discussion between the reviewers, with any differences or uncertainty resolved by consensus. The overall inter-rater reliability for the title and abstract screening will be calculated using

For peer review only - http://bmjopen.bmj.com/site/about/guidelines.xhtml

Cohen's kappa statistic.[40] After title and abstract screening is complete, all remaining full-text articles will be independently screened by KH, with any conflicts related to study inclusion resolved through discussion with RB. Where clarification is needed to determine eligibility, we will make a maximum of three attempts to contact the original study author(s).

Data extraction

Data extraction will be conducted by KH using a standardised extraction form developed by the researchers and will be checked by a second reviewer (RB). The following data will be extracted from each study: author(s), study year, country of study, study design, setting, sample size, sample characteristics (e.g., gender, age, country of origin, country/ies of transit and/or resettlement, and length of separation from parents/caregivers, where possible), reported prevalence of self-harm, method(s) used to self-harm, risk and protective factors associated with self-harm, outcomes measure(s) used, characteristics of findings including incidence or episode rates, 95% CIs, p-values and effect sizes (where reported). A maximum of three attempts will be made to contact study authors for further information if any of the required data are missing, incomplete, or unclear.

Risk of bias

The Methodological Standard for Epidemiological Research (MASTER)[41] scale will be used to assess the quality of included studies. The quality and risk of bias will be assessed by KH, with any uncertainty resolved through discussion and consensus with the second author (RB). The MASTER scale[41] provides a single consolidated tool to assess the risk of bias across different types of study design. This is done by assessing each study for the presence of several methodological standards aimed at addressing the risk of bias across six potential bias domains (selection, information, design-related, analytic, confounding, and external-validity).[41] Using the approach taken in previous systematic reviews,[42,43] we will discuss the possible risk of bias and study quality in text, as well as generate a score summarising each

For peer review only - http://bmjopen.bmj.com/site/about/guidelines.xhtml

study's quality by using the proportion of safeguards against bias that each study incorporated.

Data synthesis

We will provide a descriptive overview of the included studies, including the study year(s), design, size, and location of the study sample, measure(s) used to report self-harm, and any associated characteristics.

If a sufficient number of studies report on the rates of self-harm, we will conduct meta-analyses to calculate pooled estimates of self-harm rates. Heterogeneity will be assessed using the I^2 statistic.

If the number of included studies is sufficient, we will use meta-regression[44] to investigate the influence of gender, age, country of origin, and length of separation from parents/caregivers on rates of self-harm. To investigate the impact of study quality on risk of bias, we will conduct a sensitivity analysis which includes only papers rated as high-quality (i.e., those papers assessed as scoring above the median in the MASTER[41] scale).

If meta-analyses are not possible due to insufficient data on the rates or associated characteristics of self-harm, or if there is substantial heterogeneity, a narrative synthesis will be provided. The narrative synthesis will provide information in both text and table format to summarise and explain the included study findings. It will also explore relationships in the data, the development of a theoretical framework (if relevant), and assess the strength of the evidence for the conclusions drawn from the synthesis, as per formal guidelines on the conduct of a narrative synthesis.[45]

Patient and Public Involvement

There was no patient or public involvement in the design of this study.

Ethics and dissemination

For peer review only - http://bmjopen.bmj.com/site/about/guidelines.xhtml

For peer review only

As this is a review of studies that have already obtained ethics approval, this study is exempt from ethics approval. The findings of our review will be disseminated in a peer-reviewed journal article and via presentations at relevant national and international conferences.

Ethics statements

Patient consent for publication

Not applicable.

For peer review only

For peer review only - http://bmjopen.bmj.com/site/about/guidelines.xhtml

References

1 United Nations High Commissioner for Refugees. A record 100 million people forcibly displaced worldwide, 2022. Available: https://news.un.org/en/story/2022/05/1118772

2 United Nations High Commissioner for Refugees. Global trends report, 2021. Available: https://www.unhcr.org/62a9d1494/global-trends-report-2021

3 UNICEF. Refugee and migrant children in Europe, January – December, 2021. Available: https://www.unicef.org/eca/media/23466/file/Refugee%20and%20Migrant%20Children%20in%20Europe%20.pdf

4 UN General Assembly, Convention on the Rights of the Child, 20 November 1989, United Nations, Treaty Series, vol. 1577, p. 3. Available: https://www.refworld.org/docid/3ae6b38f0.html

5 Eurostat. First time asylum applicants up 4% in June 2022. Available: https://ec.europa.eu/eurostat/web/products-eurostat-news/-/ddn-20220926

6 Eurostat. Unaccompanied minor asylum applicants by type of applicant, citizenship, age and sex - monthly data (rounded) 30 September, 2022. Available: https://ec.europa.eu/eurostat/databrowser/view/MIGR_ASYUMACTM/default/table?lang=en&category=migr.migr_asy.migr_asyapp.migr_asyum

7 U.S. Customs and Border Protection. Southwest land border encounters 2021. Available: https://www.cbp.gov/newsroom/stats/southwest-land-border-encounters

8 Priebe S, Giacco D, El-Nagib R. Public health aspects of mental health among migrants and refugees: a review of the evidence on mental health care for refugees,

For peer review only - http://bmjopen.bmj.com/site/about/guidelines.xhtml

asylum seekers and irregular migrants in the WHO European Region. Copenhagen: WHO Regional Office for Europe; 2016. (Health Evidence Network [HEN] Synthesis Report 47). Available: https://www.ncbi.nlm.nih.gov/books/NBK391045/

9 Porter M, Haslam N. Predisplacement and postdisplacement factors associated with mental health of refugees and internally displaced persons: a meta-analysis. *JAMA* 2005;294:602-12.

10 Li SSY, Liddell BJ, Nickerson A. The relationship between post-migration stress and psychological disorders in refugees and asylum seekers. *Curr Psychiatry Rep* 2016;18:82.

11 Hodes M, Vostanis P. Practitioner Review: Mental health problems of refugee children and adolescents and their management. *J Child Psychol Psychiatry* 2019;60:716-31.

12 Vervliet M, Meyer Demott MA, Jakobsen M, Broekaert E, Heir T, Derluyn I. The mental health of unaccompanied refugee minors on arrival in the host country. *Scand J Psychol* 2014;55:33-7.

13 Bean T, Derluyn I, Eurelings-Bontekoe E, Broekaert E, Spinhoven P. Comparing psychological distress, traumatic stress reactions, and experiences of unaccompanied refugee minors with experiences of adolescents accompanied by parents. *J Nerv Ment Dis* 2007;195:288-97.

14 Noll G. Junk Science? Four arguments against the radiological age assessment of unaccompanied minors seeking asylum. *Int J Ref Law* 2016;28:234-50.

For peer review only - http://bmjopen.bmj.com/site/about/guidelines.xhtml

15 Aynsley-Green A, Cole TJ, Crawley H, Lessof N, Boag LR, Wallace RMM. Medical, statistical, ethical and human rights considerations in the assessment of age in children and young people subject to immigration control. *Br Med Bull* 2012;102:17-42.

16 European Commission, Directorate-General for Justice and Consumers. EU strategy on the rights of the child, 2021. Available: https://data.europa.eu/doi/10.2838/313794

17 von Werthern M, Grigorakis G, Vizard E. The mental health and wellbeing of Unaccompanied Refugee Minors (URMs). *Child Abuse Negl* 2019;98:104146.

18 Derluyn I, Mels C, Broekaert E. Mental health problems in separated refugee adolescents. *J Adolesc Health* 2009;44:291-7.

19 Kirk Felsman J, Leong FTL, Johnson MC, Crabtree Felsman I. Estimates of psychological distress among Vietnamese refugees: Adolescents, unaccompanied minors and young adults. *Soc. Sci. Med* 1990;3:1251-6.

20 Hodes M, Jagdev D, Chandra N, Cunniff A. Risk and resilience for psychological distress amongst unaccompanied asylum seeking adolescents. *J Child Psychol Psychiatry* 2008;49:723-32.

21 Pinto Wiese EB, Burhorst I. The mental health of asylum-seeking and refugee children and adolescents attending a clinic in the Netherlands. *Transcult Psychiatry* 2007;44:596-613.

22 Bamford J, Fletcher M, Leavey G. Mental health outcomes of unaccompanied refugee minors: a rapid review of recent research. *Curr Psychiatry Rep* 2021;23(8):46.

For peer review only

For peer review only - http://bmjopen.bmj.com/site/about/guidelines.xhtml

23 Höhne E, van der Meer AS, Kamp-Becker I, Christiansen H. A systematic review of risk and protective factors of mental health in unaccompanied minor refugees. *Eur Child Adolesc Psychiatry* 2022;3:1-15.

24 Huemer J, Karnik NS, Voelkl-Kernstock S, Granditsch E, Dervic K, Friedrich MH, et al. Mental health issues in unaccompanied refugee minors. *Child Adolesc Psychiatry Ment Health* 2009;3:13.

25 Fazel M, Reed RV, Panter-Brick C, Stein A. Mental health of displaced and refugee children resettled in high-income countries: risk and protective factors. *Lancet* 2012;379:266-82.

26 Gargiulo A, Tessitore F, Le Grottaglie F, Margherita G. Self-harming behaviours of asylum seekers and refugees in Europe: A systematic review. *Int J Psychol* 2021;56:189-98.

27 Cohen J. Safe in our hands?: A study of suicide and self-harm in asylum seekers. *J Forensic Leg Med* 2008;15:235-44.

28 Fazel S, Runeson B. Suicide. *N Engl J Med* 2020;382:266-74.

29 Ramel B, Täljemark J, Lindgren A, Johansson B. Overrepresentation of unaccompanied refugee minors in inpatient psychiatric care. *Springerplus* 4: 131. 2015.

30 Daniel-Calveras A, Baldaqui N, Baeza I. Mental health of unaccompanied refugee minors in Europe: A systematic review. *Child Abuse Negl* 2022;133:105865.

31 Armitage AJ, Cohen J, Heys M, Hardelid P, Ward A, Eisen S. Description and evaluation of a pathway for unaccompanied asylum-seeking children. *Arch Dis Child* 2022;107:456-60.

For peer review only

For peer review only - http://bmjopen.bmj.com/site/about/guidelines.xhtml

32 Edmondson AJ, Brennan CA, House AO. Non-suicidal reasons for self-harm: A systematic review of self-reported accounts. *J Affect Disord* 2016;191:109-17.

33 Moher D, Shamseer L, Clarke M, *et al*. Preferred reporting items for systematic review and meta-analysis protocols (PRISMA-P) 2015 statement. *Syst Rev* 2015;4:1.

34 United Nations High Commissioner for Refugees. Convention and Protocol Relating to the Status of Refugees, 2010. Available: http://www.unhcr.org/3b66c2aa10.html

35 Phillip J. Asylum seekers and refugees. What are the facts? 2015. Available: https://www.aph.gov.au/About_Parliament/Parliamentary_Departments/Parliamentary_Library/pubs/rp/rp1415/AsylumFacts

36 Hawton K, Harriss L, Hall S, *et al*. Deliberate self-harm in Oxford, 1990–2000: a time of change in patient characteristics. *Psychol Med* 2003;33:987-95.

37 World Health Organization. Classification of diseases (ICD), 2018. Available: http://www.who.int/classifications/icd/en/

38 Clarivate Analytics. Endnote X8, 2018. Available: https://endnote. com/ [accessed 8 September 2022].

39 Covidence. Available: https://www.covidence.org/ [accessed 5 Oct 2022].

40 McHugh ML. Interrater reliability: the kappa statistic. *Biochem Med* 2012;22:276-82.

41 Stone JC, Glass K, Clark J, *et al.* A unified framework for bias assessment in clinical research. *Int J Evid Based Healthc* 2019;17:106-20.

42 Borschmann R, Janca E, Carter A, *et al*. The health of adolescents in detention: a global scoping review. *Lancet Public Health* 2020;5:e114-26.

For peer review only

For peer review only - http://bmjopen.bmj.com/site/about/guidelines.xhtml

43 Willoughby M, Young JT, Spittal MJ, *et al*. Violence-related deaths among people released from incarceration: systematic review and meta-analysis of cohort studies. *E Clin Med* 2021;41:101162.

44 Baker WL, Michael White C, Cappelleri JC, *et al*. Understanding heterogeneity in meta-analysis: the role of meta-regression. *Int J Clin Pract* 2009;63:1426-34.

45 Popay J, Roberts H, Sowden A, *et al*. Guidance on the conduct of narrative synthesis in systematic reviews: a product from the ESRC methods program, 2006. Available: https://www.lancaster.ac.uk/media/lancaster-university/content-assets/documents/fhm/dhr/chir/NSsynthesisguidanceVersion1-April2006.pdf

Authors' contributions: KH developed the original research proposal. KH and RB both contributed to the design of the project. KH developed the search strategy, with input from RB. KH wrote the initial draft of the manuscript, and RB contributed significantly to drafting and editing the manuscript. All authors approved the final manuscript.

Funding: This research received no specific grant from any funding agency in the public, commercial, or not-for-profit sectors. RB receives salary and research support from a National Health and Medical Research Council Emerging Leadership Investigator Grant (EL2; GNT2008073).

Competing interests: None declared.

For peer review only

For peer review only - http://bmjopen.bmj.com/site/about/guidelines.xhtml

PRISMA-P 2015 Checklist

This checklist has been adapted for use with protocol submissions to *Systematic Reviews* from Table 3 in Moher D et al: Preferred reporting items for systematic review and meta-analysis protocols (PRISMA-P) 2015 statement. *Systematic Reviews* 2015 4:1

Section/topic	#	Checklist item	Information reported		Line number(s)
			Yes	No	
**ADMINISTRATIVE INFORMATION**					
**Title**					
Identification	1a	Identify the report as a protocol of a systematic review	X		p.1
Update	1b	If the protocol is for an update of a previous systematic review, identify as such			N/A
**Registration**	2	If registered, provide the name of the registry (e.g., PROSPERO) and registration number in the Abstract	X		p.3 p.7
**Authors**					
Contact	3a	Provide name, institutional affiliation, and e-mail address of all protocol authors; provide physical mailing address of corresponding author	X		p.1
Contributions	3b	Describe contributions of protocol authors and identify the guarantor of the review	X		p.18
**Amendments**	4	If the protocol represents an amendment of a previously completed or published protocol, identify as such and list changes; otherwise, state plan for documenting important protocol amendments			N/A
**Support**					
Sources	5a	Indicate sources of financial or other support for the review	X		p.18
Sponsor	5b	Provide name for the review funder and/or sponsor			N/A
Role of sponsor/funder	5c	Describe roles of funder(s), sponsor(s), and/or institution(s), if any, in developing the protocol	X		p.18
**INTRODUCTION**					
**Rationale**	6	Describe the rationale for the review in the context of what is already known	X		pp.4-7
**Objectives**	7	Provide an explicit statement of the question(s) the review will address with reference to participants, interventions, comparators, and outcomes (PICO)	X		pp.6-7

BioMed Central The Open Access Publisher

Section/topic	#	Checklist item	Information reported Yes	Information reported No	Line number(s)
**METHODS**					
**Eligibility criteria**	8	Specify the study characteristics (e.g., PICO, study design, setting, time frame) and report characteristics (e.g., years considered, language, publication status) to be used as criteria for eligibility for the review	X	☐	pp.7-8
**Information sources**	9	Describe all intended information sources (e.g., electronic databases, contact with study authors, trial registers, or other grey literature sources) with planned dates of coverage	X	☐	pp.9-10
**Search strategy**	10	Present draft of search strategy to be used for at least one electronic database, including planned limits, such that it could be repeated	X	☐	p.9
STUDY RECORDS					
Data management	11a	Describe the mechanism(s) that will be used to manage records and data throughout the review	X	☐	pp.8-9
Selection process	11b	State the process that will be used for selecting studies (e.g., two independent reviewers) through each phase of the review (i.e., screening, eligibility, and inclusion in meta-analysis)	X	☐	pp.9-10
Data collection process	11c	Describe planned method of extracting data from reports (e.g., piloting forms, done independently, in duplicate), any processes for obtaining and confirming data from investigators	X	☐	p.10
**Data items**	12	List and define all variables for which data will be sought (e.g., PICO items, funding sources), any pre-planned data assumptions and simplifications	X	☐	p. 8 pp.10-11
**Outcomes and prioritization**	13	List and define all outcomes for which data will be sought, including prioritization of main and additional outcomes, with rationale	X	☐	pp.10-11
**Risk of bias in individual studies**	14	Describe anticipated methods for assessing risk of bias of individual studies, including whether this will be done at the outcome or study level, or both; state how this information will be used in data synthesis	X	☐	pp.10-11
DATA					
**Synthesis**	15a	Describe criteria under which study data will be quantitatively synthesized	X	☐	pp.10-11
	15b	If data are appropriate for quantitative synthesis, describe planned summary measures, methods of handling data, and methods of combining data from studies, including any planned exploration of consistency (e.g., I^2, Kendall's tau)	X	☐	p.11
	15c	Describe any proposed additional analyses (e.g., sensitivity or subgroup analyses, meta-regression)	X	☐	p.11
	15d	If quantitative synthesis is not appropriate, describe the type of summary planned	X	☐	p.11
**Meta-bias(es)**	16	Specify any planned assessment of meta-bias(es) (e.g., publication bias across studies, selective	X	☐	p.11

BioMed Central The Open Access Publisher

Section/topic	#	Checklist item	Information reported		Line number(s)
			Yes	No	
		reporting within studies)			
**Confidence in cumulative evidence**	17	Describe how the strength of the body of evidence will be assessed (e.g., GRADE)	X	☐	p.11

BioMed Central
The Open Access Publisher

BMJ Open

Self-harm among unaccompanied asylum seekers and refugee minors: protocol for a global systematic review of prevalence, methods, and characteristics

Journal:	*BMJ Open*
Manuscript ID	bmjopen-2022-069237.R1
Article Type:	Protocol
Date Submitted by the Author:	01-Mar-2023
Complete List of Authors:	Hedrick, Kyli; Uppsala Universitet, Department of Public Health and Caring Sciences; The University of Melbourne - Parkville Campus, Centre for Health Equity, Melbourne School of Population and Global Health<br>Borschmann, Rohan; The University of Melbourne, Centre for Health Equity, Melbourne School of Population and Global Health; Oxford University, Department of Psychiatry, Warneford Hospital
**Primary Subject Heading**:	Mental health
Secondary Subject Heading:	Public health
Keywords:	MENTAL HEALTH, PUBLIC HEALTH, Suicide & self-harm < PSYCHIATRY, Child & adolescent psychiatry < PSYCHIATRY

SCHOLARONE™
Manuscripts

Self-harm among unaccompanied asylum seekers and refugee minors: protocol for a global systematic review of prevalence, methods, and characteristics

Kyli Hedrick[1,2], Rohan Borschmann[2,3,4,5]

[1]Child Health and Parenting (CHAP), Department of Public Health and Caring Sciences, Uppsala University, BMC, Husargatan 3, 753 27, Uppsala, Sweden.

[2]Centre for Mental Health; Melbourne School of Population and Global Health, The University of Melbourne, Melbourne, Victoria, 3010, Australia

[3]Department of Psychiatry; University of Oxford, Warneford Hospital, Oxford OX3 7JX, UK

[4]Centre for Adolescent Health; Murdoch Children's Research Institute, Melbourne, Victoria, 3052, Australia

[5]Melbourne School of Psychological Sciences; University of Melbourne, Melbourne, Victoria, 3010, Australia

[6]School of Population Health, Curtin University, Western Australia, 6845, Australia

Corresponding author: Dr. Kyli Hedrick

Email: kyli.hedrick@pubcare.uu.se

For peer review only

For peer review only - http://bmjopen.bmj.com/site/about/guidelines.xhtml

Abstract

***Introduction*:** Asylum seekers and refugees are at an elevated risk of self-harm, with younger age and traumatic experiences found to further increase such risk. Despite this, evidence regarding self-harm among unaccompanied asylum seekers and refugee minors has not been synthesised. As self-harm among minors is a risk factor for a range of adverse clinical and social outcomes, including suicide, such information may help to inform evidence-based prevention strategies among these vulnerable populations. This systematic review will synthesise findings from the literature regarding the prevalence, methods, and characteristics of self-harm, including risk and protective factors, among unaccompanied asylum seekers and refugee minors internationally.

***Methods and analysis*:** We searched key electronic health, psychology, and medical databases (PsycINFO, Scopus, PubMed, and Ovid MEDLINE) and grey literature for relevant studies published in English from database inception to 10 February 2023. Our primary outcome is self-harm among unaccompanied asylum seekers and/or refugee minors. We will include all types of study design (except single case studies) that examine the prevalence of self-harm in unaccompanied asylum seekers and/or refugee minors. We will exclude dissertations, conference abstracts, letters, book chapters, editorials, study registrations, clinical trials, case-control studies, registered protocols, and qualitative studies. Only studies reporting on participants aged <18 years will be eligible for inclusion. The Methodological Standard for Epidemiological Research (MASTER) scale will be used to assess the quality of included studies. If there are sufficient studies, and homogeneity between them, we will conduct meta-analyses to calculate pooled estimates of self-harm rates, as well as comparisons between sub-groups of relevance. If the studies do not report sufficient data, or there is substantial heterogeneity, we will provide a narrative synthesis of the findings.

For peer review only - http://bmjopen.bmj.com/site/about/guidelines.xhtml

Ethics and dissemination: This review is exempt from ethics approval. Our findings will be disseminated through a peer-reviewed publication and conference presentations.

PROSPERO registration number CRD42021292709.

Strengths and limitations of this study

- This systematic review uses a comprehensive search strategy including four key academic databases and a grey literature search.
- A strength of this study is that only studies involving unaccompanied asylum seekers and refugee minors and other immigrant populations, and that distinguish between these populations (i.e., consider these populations to be heterogenous), will be included.
- Studies that examine self-harm and suicide attempts but do not distinguish between these acts will be excluded as these two outcomes are qualitatively and motivationally distinct from one another.
- A small number of primary studies, and heterogeneity between study populations and/or study design, may preclude meta-analysis or direct comparison between studies.

For peer review only

For peer review only - http://bmjopen.bmj.com/site/about/guidelines.xhtml

Introduction

Globally, the number of people forcibly displaced because of war, conflict, and political unrest has reached record highs. According to the United Nations High Commissioner for Refugees (UNHCR) [1], as at the end of May 2022, there are now over 100 million forcibly displaced people around the world. Children and young people are markedly over-represented in this humanitarian crisis, accounting for an estimated 41% of those displaced, though they make up 30% of the world's population [2]. Of the 24,100 children who arrived in Greece, Italy, Bulgaria, Spain, Cyprus, and Malta in the 12-month period to December 2021, 17,200 (71%) were unaccompanied and separated [3]. The UNHCR defines a refugee as a person who: 1) has a well-founded fear of being persecuted for reasons relating to race, religion, nationality, membership of a particular social group, or political opinion; 2) is outside the country of (their) nationality; and 3) is unable, or owing to such fear, is unwilling, to avail (themselves) of the protection of that country (p.14) [4]. An asylum seeker is defined as an individual who is seeking refugee 'protection, but whose claim for refugee status has not yet been determined' [5]. Unaccompanied asylum seeker and refugee minors are defined by the United Nations Convention on the Rights of the Child [6] as children or adolescents younger than 18 years who have been separated from both parents and relatives and are not being cared for by an adult, who, by law or custom, is responsible for doing so.

First-time asylum applications from unaccompanied minors in the European Union (EU) Member States with available data increased by 10% between May and June 2022, and 63% between June 2021 and June 2022 [7]. Whilst most unaccompanied minors are males aged between 14-17 years, numbers of female and younger populations are increasing [8]. In 2022, the main countries of origin for unaccompanied minors in the EU Member States were Afghanistan, Syria, Somalia, and Pakistan [7]. In the 2021 financial year, the United States

For peer review only

(US) reported receiving an all-time record of unaccompanied minors from Central America at or near the US-Mexico border, including 145,000 minors [9].

Many individuals who have been forcibly displaced have been subjected to a range of potentially traumatic events in the pre-, peri-, and post-migration phases [10]. These include witnessing the death of a family member or loved one, war, dangerous flight, poor humanitarian conditions, and periods of detention [10, 11], followed by the challenges associated with managing the uncertainty of the asylum process, and the resettlement process [12]. Young people who have been forcibly displaced must navigate such adverse experiences during a challenging developmental period, which may further compound the detrimental impact of these events on their mental health [13]. For young people who are forcibly displaced without parents or caregivers, they must not only navigate several potentially traumatic experiences during a difficult time developmentally, but they must do so without the support and guidance of a parent or caregiver, and whilst managing the loss associated with separation from parents and/or family [14]. Upon arrival in host countries, unaccompanied minors may also be confronted with persistent questioning by authorities about their age, familial relationships, and reason(s) for leaving their home country [15]. In addition, they may be subjected to invasive and imprecise age assessments such as carpal, collarbone, and/or dental x-rays to establish the accuracy of their self-reported age [16, 17]. The particular vulnerabilities and risks faced by unaccompanied asylum seekers and refugee minors have been increasingly acknowledged, paving the way for their entitlement to international protection [6], as well as programs promoting their physical, mental, and educational development [6, 18].

Research has documented the numerous adverse mental health consequences for unaccompanied asylum seekers and refugee minors across several settings and locations [19]. For example, a 2019 global systematic review of the mental health of unaccompanied refugee

For peer review only - http://bmjopen.bmj.com/site/about/guidelines.xhtml

minors by von Wethern and colleagues [19] found that despite varying estimates of psychological difficulties, rates of PTSD (17-85%), depression (13-76%), and anxiety (11-85%) were significantly higher in unaccompanied refugee minors than those found in the general western population. Evidence compiled by von Werthern et al. [19] also found that adolescence and being female were indicators of increased risk of psychiatric disorders. In addition, the authors' review highlighted that there was significantly more exposure to traumatic events such as physical violence, torture, imprisonment, and killings among unaccompanied minors, compared with exposure in the general population [20, 21, 22, 23]. Earlier systematic reviews have largely focused on the mental health and wellbeing of unaccompanied minors [19, 24, 25, 26], or the mental health of refugee minors [27]. However, preliminary searches conducted by the authors for the purposes of this study (in PROSPERO, the PUBMED/MEDLINE and DARE databases, and the JBI Evidence Synthesis journal [28]) indicate that no reviews have examined self-harm (elsewhere also described as non-suicidal self-injury [NSSI], and defined as the deliberate, self-inflicted destruction of body tissue, without suicidal intent, and for purposes not socially or culturally sanctioned [29]) in unaccompanied asylum seekers and refugee minors globally. As self-harm is common among young people [30, 31], and this risk is further elevated among younger asylum seekers and refugees and those with experiences of trauma [32, 33], it is conceivable that unaccompanied asylum seekers and refugee minors are at even greater risk of self-harm. Should a higher prevalence of self-harm among unaccompanied asylum seekers and refugee minors be identified, these findings would elucidate the need for providing mental health support at the earliest available opportunity for such minors. Indeed, given that every act of self-harm (including explicitly non-suicidal actions and behaviour) has the potential to be lethal as a result of accident or misfortune, self-harm (irrespective of intent) is an extremely important outcome to target. Furthermore, as the social and public health costs of self-harm

For peer review only - http://bmjopen.bmj.com/site/about/guidelines.xhtml

are known to be high [34, 35], the synthesis of such evidence may help to inform distribution of scarce public resources. One broader review that focused on self-harming behaviours among immigrants in Europe by Garguilo and colleagues [36] did report – via a synthesis of three pertinent studies - that unaccompanied minors were an 'at-risk' group for self-harm in the European context. However, as unaccompanied minors were not explicitly included in Garguilo and colleagues' [36] search strategy, and the setting of their review was European, rather than global, it is likely that other salient studies may have been excluded. As such, a comprehensive global review synthesising the evidence relating to self-harm among unaccompanied asylum seekers and refugee minors could inform evidence-based prevention and management strategies in these vulnerable populations.

A Swedish study by Ramel and colleagues [37] was included in both von Werthern and colleagues [19] and Garguilo and colleagues [36] reviews. In this study, the authors compared the psychiatric care of unaccompanied refugee minors and accompanied refugee minors (with an average age of 15.1 years), reporting that 3.4% (or 56/1,657) of unaccompanied refugee minors received inpatient psychiatric care and 0.67% involuntary care, compared to 0.26% (205/81,077) and 0.02% respectively of the accompanied refugee minors [37]. Both comparisons were statistically significant ($p < 0.001$). In addition, Ramel and colleagues found that a higher proportion of unaccompanied refugee minors (21.8; 76%) than accompanied minors (21.1; 56%) exhibited self-harm or suicidal behaviour on admission to hospital ($p < 0.001$) [37]. The study also noted that 86% (13.2) of unaccompanied minors were hospitalised due to the stress associated with the asylum process [37]. A 2022 systematic review of the mental health of unaccompanied minors in Europe by Daniel-Calveras and colleagues [38] also included one retrospective study evaluating data from 101 unaccompanied refugee minors (aged 14-17) presenting to health care providers in the United Kingdom, reporting that 8% (8/101) had engaged in self-harm or suicidal behaviour [39].

For peer review only - http://bmjopen.bmj.com/site/about/guidelines.xhtml

None of the studies included in the reviews by von Werthern [19], Garguilo [36], or Daniel-Calveras and colleagues [38] (or the original authors of the two studies highlighted above [37, 39]), however, separated out or distinguished – where possible – episodes of self-harm from suicidal behaviour when presenting their findings, though Garguilo and colleagues [36] did state in their discussion that self-harm should be viewed as a distinct clinical entity. Indeed, definitions of self-harming behaviour have varied historically [40, 41], and behavioural intent is difficult to measure. Despite this, as self-harm and suicide attempts are motivationally and qualitatively distinct from one another [42], differentiating between the two – where possible – is critical to inform the provision of appropriate physical and mental health care. Furthermore, as no terms relating to self-harm were included in von Werthern and colleagues' [19] or Daniel-Calveras and colleagues' [38] search strategies, and those included in Garguilo et al.'s [36] search strategy were not exhaustive, additional studies focusing specifically on self-harm (as distinct from suicidal behaviour) may not have been identified in these reviews.

We aim to synthesise the evidence regarding the prevalence, methods, and characteristics of self-harm, including risk and protective factors, among unaccompanied asylum seekers and refugee minors internationally.

Methods and analysis

This protocol is reported in accordance with the Preferred Reporting Items for Systematic Reviews and Meta-Analysis Protocols (PRISMA-P) [43].

Patient and Public Involvement

There was no patient or public involvement in the design of this study.

Eligibility criteria

Participants

For peer review only - http://bmjopen.bmj.com/site/about/guidelines.xhtml

We will include studies examining self-harm among unaccompanied asylum seekers and refugee minors – defined as a person aged <18 years who has been separated from both parents and relatives and is not being cared for by an adult, who, by law or custom, is responsible for doing so [6] – in any country. Studies reporting on unaccompanied asylum seekers and/or refugee minors and other immigrant populations, but that do not distinguish between these populations, will be excluded. Studies reporting on suicide or suicide attempts only will be excluded. Where studies refer to suicide and self-harm separately, only findings regarding self-harm will be included. Comparison studies involving accompanied asylum seekers and/or refugee minors, as well and the general/native population in countries of origin, transit, and/or resettlement will also be included, where available.

Outcome measure

Our primary outcome measure will be self-harm. For the purposes of this review, self-harm, elsewhere also described as non-suicidal self-injury [NSSI], is defined as the deliberate, self-inflicted destruction of body tissue, without suicidal intent, and for purposes not socially or culturally sanctioned [29]. It may include behaviours such as cutting, burning, biting, and scratching skin [29]. Self-harm may be measured by self-report, clinical interview, or administrative data (e.g., emergency department [ED], hospital, or incident reports). An additional outcome measure will be method(s) used to self-harm. Methods of self-harm may also be measured by self-report, clinical interview, or administrative data, including the World Health Organization's International Classification of Diseases Tenth Revision (ICD-10) codes [44].

Study design

We will include published cohort studies (prospective and retrospective) of unaccompanied asylum seekers and refugee minors which report on the prevalence of self-harm. We will also

For peer review only - http://bmjopen.bmj.com/site/about/guidelines.xhtml

include grey literature (see "Information sources and search strategy" below). We will exclude single case studies, dissertations, conference abstracts, letters, book chapters, editorials, study registrations, clinical trials, case-control studies, registered protocols, and qualitative studies. We will not include previous systematic reviews, as not all included studies may not meet our inclusion criteria. We will, however, identify any peer-reviewed studies related to any dissertations, conference abstracts, study registrations, and studies assessed for previous reviews that meet our inclusion criteria but were not identified via our search strategy. Only studies published in English will be included.

Information sources and search strategy

We searched four key health and medical databases (PsycINFO, Scopus, PubMed, and OVID MEDLINE) for relevant literature published in English using key terms relating to self-harm, migration, and minors, from database inception until the date of our final search (10 February 2023). The MEDLINE search strategy is outlined in table 1. The full search strategy for each database is outlined in Online supplemental appendix 1.The electronic database searches will be supplemented by reviewing the reference lists of eligible articles, as well as searching the websites of relevant non-governmental organisations who have worked with unaccompanied asylum seekers and refugee minors (such as Médecins sans Frontières and Save the Children), for pertinent grey literature.

Table 1

MEDLINE search strategy

1.	(non-suicidal self-harm or self-injur* or deliberate self-harm or self-poison* or self-mutilat*).af.
2.	(migration or immigration or unaccompanied asylum seeker or unaccompanied refugee minor or child or adolescent or teenager).af.
3.	1 and 2

Study selection

For peer review only - http://bmjopen.bmj.com/site/about/guidelines.xhtml

All studies identified through the database search will be downloaded to Endnote [45] and duplicates removed. The remaining studies will be imported into Covidence [46] for screening. All titles and abstracts will be screened for inclusion by the primary author (KH), with 20% screened by the second author (RB). After 20% of the papers identified in the search strategy have been double screened, we will reassess our eligibility criteria to ensure that they are relevant to the studies that are identified. The reassessment process will involve a discussion between the reviewers, with any differences or uncertainty regarding study inclusion resolved by a third researcher. The overall inter-rater reliability for the title and abstract screening will be calculated using Cohen's kappa statistic [47]. Studies will be coded as either 0=Does not meet eligibility criteria, or 1=Meets eligibility criteria or the full-text article needs to be screened to confirm eligibility. After 20% of the citations have been screened, the eligibility criteria will be reassessed through discussion between all researchers to ensure that they are relevant to the studies that have been identified. If applicable, the updated eligibility criteria will be used for the remaining screening.

After title and abstract screening is complete, all remaining full-text articles will be independently screened by KH and RB, with any conflicts related to study inclusion resolved through discussion with a third researcher. Where clarification is needed to determine eligibility, we will make a maximum of three attempts to contact the original study author(s), with no more attempts at contact made after two months.

Data extraction

Data extraction will be conducted by KH using a standardised extraction form developed by the researchers and will be checked by a second reviewer (RB). The following data will be extracted from each study: author(s), study year, country of study, study design, setting, sample size, sample characteristics (e.g., gender, age, country of origin, country/ies of transit and/or resettlement, and length of separation from parents/caregivers, where possible),

For peer review only - http://bmjopen.bmj.com/site/about/guidelines.xhtml

reported prevalence of self-harm, method(s) used to self-harm, risk and protective factors associated with self-harm, outcomes measure(s) used, characteristics of findings including incidence or episode rates, 95% CIs, p-values and effect sizes (where reported). A maximum of three attempts will be made to contact study authors for further information if any of the required data are missing, incomplete, or unclear.

Risk of bias

The Methodological Standard for Epidemiological Research (MASTER) [48] scale will be used to assess the quality of included studies. The quality and risk of bias will be assessed by KH, with any uncertainty resolved through discussion and consensus with the second author (RB). The MASTER scale [48] provides a single consolidated tool to assess the risk of bias across different types of study design. This is done by assessing each study for the presence of several methodological standards aimed at addressing the risk of bias across six potential bias domains (selection, information, design-related, analytic, confounding, and external-validity) [48]. Using the approach taken in previous systematic reviews [49, 50], we will discuss the possible risk of bias and study quality in text, as well as generate a score summarising each study's quality by using the proportion of safeguards against bias that each study incorporated.

Data synthesis

We will provide a descriptive overview of the included studies, including the study year(s), design, size, and location of the study sample, measure(s) used to report self-harm, and any associated characteristics.

If a sufficient number of studies report on the rates of self-harm, or associated characteristics of self-harm, and there is sufficient homogeneity between studies (by, for example, design, sample, or outcome), we will conduct meta-analyses to calculate pooled estimates of self-harm rates. Heterogeneity will be assessed using the I^2 statistic.

For peer review only - http://bmjopen.bmj.com/site/about/guidelines.xhtml

If the number of included studies is sufficient, we will use meta-regression [51] to investigate the influence of gender, age, country of origin, and length of separation from parents/caregivers on rates of self-harm. To investigate the impact of study quality on risk of bias, we will conduct a sensitivity analysis which includes only papers rated as high-quality (i.e., those papers assessed as scoring above the median in the MASTER [48] scale).

If meta-analyses are not possible due to insufficient data on the rates or associated characteristics of self-harm, or if there is substantial heterogeneity, a narrative synthesis will be created (as per established guidelines on the conduct of a narrative synthesis [52]). This descriptive overview will provide information in both text and table format to summarise and explain the included study findings, based on outcome, associated characteristics of self-harm, and geographic region. From this, we may also develop a theory [52] of self-harm among unaccompanied asylum seekers and refugee minors, and the factors that influence it, and an assessment of the overall robustness of this summary.

Ethics and dissemination

As this is a review of studies that have already obtained ethics approval, this study is exempt from ethics approval. The findings of our review will be disseminated in a peer-reviewed journal article and via presentations at relevant national and international conferences.

Ethics statements

Patient consent for publication

Not applicable.

For peer review only

References

1 United Nations High Commissioner for Refugees. A record 100 million people forcibly displaced worldwide, 2022. Available: https://news.un.org/en/story/2022/05/1118772

2 United Nations High Commissioner for Refugees. Global trends report, 2021. Available: https://www.unhcr.org/62a9d1494/global-trends-report-2021

3 UNICEF. Refugee and migrant children in Europe, January – December, 2021. Available: https://www.unicef.org/eca/media/23466/file/Refugee%20and%20Migrant%20Children%20in%20Europe%20.pdf

4 United Nations High Commissioner for Refugees. Convention and Protocol Relating to the Status of Refugees, 2010. Available: http://www.unhcr.org/3b66c2aa10.html

5 Phillip J. Asylum seekers and refugees. What are the facts? 2015. Available: https://www.aph.gov.au/About_Parliament/Parliamentary_Departments/Parliamentary_Library/pubs/rp/rp1415/AsylumFacts

6 UN General Assembly, Convention on the Rights of the Child, 20 November 1989, United Nations, Treaty Series, vol. 1577, p. 3. Available: https://www.refworld.org/docid/3ae6b38f0.html

7 Eurostat. First time asylum applicants up 4% in June 2022. Available: https://ec.europa.eu/eurostat/web/products-eurostat-news/-/ddn-20220926

8 Eurostat. Unaccompanied minor asylum applicants by type of applicant, citizenship, age and sex - monthly data (rounded) 30 September, 2022. Available:

For peer review only

https://ec.europa.eu/eurostat/databrowser/view/MIGR_ASYUMACTM/default/table?lang=en&category=migr.migr_asy.migr_asyapp.migr_asyum

9 U.S. Customs and Border Protection. Southwest land border encounters 2021. Available: https://www.cbp.gov/newsroom/stats/southwest-land-border-encounters

10 Priebe S, Giacco D, El-Nagib R. Public health aspects of mental health among migrants and refugees: a review of the evidence on mental health care for refugees, asylum seekers and irregular migrants in the WHO European Region. Copenhagen: WHO Regional Office for Europe; 2016. (Health Evidence Network [HEN] Synthesis Report 47). Available: https://www.ncbi.nlm.nih.gov/books/NBK391045/

11 Porter M, Haslam N. Predisplacement and postdisplacement factors associated with mental health of refugees and internally displaced persons: a meta-analysis. *JAMA* 2005;294:602-12.

12 Li SSY, Liddell BJ, Nickerson A. The relationship between post-migration stress and psychological disorders in refugees and asylum seekers. *Curr Psychiatry Rep* 2016;18:82.

13 Hodes M, Vostanis P. Practitioner Review: Mental health problems of refugee children and adolescents and their management. *J Child Psychol Psychiatry* 2019;60:716-31.

14 Vervliet M, Meyer Demott MA, Jakobsen M, Broekaert E, Heir T, Derluyn I. The mental health of unaccompanied refugee minors on arrival in the host country. *Scand J Psychol* 2014;55:33-7.

15 Bean T, Derluyn I, Eurelings-Bontekoe E, Broekaert E, Spinhoven P. Comparing psychological distress, traumatic stress reactions, and experiences of unaccompanied

For peer review only - http://bmjopen.bmj.com/site/about/guidelines.xhtml

refugee minors with experiences of adolescents accompanied by parents. *J Nerv Ment Dis* 2007;195:288-97.

16 Noll G. Junk Science? Four arguments against the radiological age assessment of unaccompanied minors seeking asylum. *Int J Ref Law* 2016;28:234-50.

17 Aynsley-Green A, Cole TJ, Crawley H, Lessof N, Boag LR, Wallace RMM. Medical, statistical, ethical and human rights considerations in the assessment of age in children and young people subject to immigration control. *Br Med Bull* 2012;102:17-42.

18 European Commission, Directorate-General for Justice and Consumers. EU strategy on the rights of the child, 2021. Available: https://data.europa.eu/doi/10.2838/313794

19 von Werthern M, Grigorakis G, Vizard E. The mental health and wellbeing of Unaccompanied Refugee Minors (URMs). *Child Abuse Negl* 2019;98:104146.

20 Derluyn I, Mels C, Broekaert E. Mental health problems in separated refugee adolescents. *J Adolesc Health* 2009;44:291-7.

21 Kirk Felsman J, Leong FTL, Johnson MC, Crabtree Felsman I. Estimates of psychological distress among Vietnamese refugees: Adolescents, unaccompanied minors and young adults. *Soc. Sci. Med* 1990;3:1251-6.

22 Hodes M, Jagdev D, Chandra N, Cunniff A. Risk and resilience for psychological distress amongst unaccompanied asylum seeking adolescents. *J Child Psychol Psychiatry* 2008;49:723-32.

23 Pinto Wiese EB, Burhorst I. The mental health of asylum-seeking and refugee children and adolescents attending a clinic in the Netherlands. *Transcult Psychiatry* 2007;44:596-613.

For peer review only - http://bmjopen.bmj.com/site/about/guidelines.xhtml

24 Bamford J, Fletcher M, Leavey G. Mental health outcomes of unaccompanied refugee minors: a rapid review of recent research. *Curr Psychiatry Rep* 2021;23(8):46.

25 Höhne E, van der Meer AS, Kamp-Becker I, Christiansen H. A systematic review of risk and protective factors of mental health in unaccompanied minor refugees. *Eur Child Adolesc Psychiatry* 2022;3:1-15.

26 Huemer J, Karnik NS, Voelkl-Kernstock S, Granditsch E, Dervic K, Friedrich MH, et al. Mental health issues in unaccompanied refugee minors. *Child Adolesc Psychiatry Ment Health* 2009;3:13.

27 Fazel M, Reed RV, Panter-Brick C, Stein A. Mental health of displaced and refugee children resettled in high-income countries: risk and protective factors. *Lancet* 2012;379:266-82.

28 Munn Z, Moola S, Lisy K, Riitano D, Tufanaru C. Chapter 5: Systematic reviews of prevalence and incidence. In: Aromataris E, Munn Z (Editors). *JBI Manual for Evidence Synthesis.* JBI, 2020. Available: https://synthesismanual.jbi.global. https://doi.org/10.46658/JBIMES-20-06

29 International Society for the Study of Self-Injury. Definition of non-suicidal self-injury, 2014. Available: https://www.itriples.org/what-is-nssi.

30 Moran, P., Coffey, C., Romaniuk, H., Olsson, C., Borschmann, R., Carlin, J.B., & Patton, G.C. (2011). The natural history of self-harm during adolescence and young adulthood: population-based cohort study. *The Lancet*, *379(9812)*, 236-243.

31 Borschmann, R, Mundy, LK, Canterford, L, Moreno-Betancur, M, Moran, PA, Viner, RM, Allen, NB, Degenhardt, L, Kosoloa, S, Fedyzsyn, I, Patton, GC. (2020). Self-

For peer review only - http://bmjopen.bmj.com/site/about/guidelines.xhtml

harm in primary school-aged children: prospective cohort study. *PLOS ONE* (in press).

32 Cohen J. Safe in our hands?: A study of suicide and self-harm in asylum seekers. *J Forensic Leg Med* 2008;15:235-44.

33 Fazel S, Runeson B. Suicide. *N Engl J Med* 2020;382:266-74.

34 Mehlum L. Cost of self-harm to society is high and increasing: a call for evidence-based and systematic treatment approaches. *Acta Psychiatrica Scandinavica*. 2022;145(4):317-8.

35 Mitchell RJ, Seah R, Ting HP, Curtis K, Foster K. Intentional self-harm and assault hospitalisations and treatment cost of children in Australia over a 10-year period. *Australian and New Zealand Journal of Public Health*. 2018;42(3):240-6.

36 Gargiulo A, Tessitore F, Le Grottaglie F, Margherita G. Self-harming behaviours of asylum seekers and refugees in Europe: A systematic review. *Int J Psychol* 2021;56:189-98.

37 Ramel B, Täljemark J, Lindgren A, Johansson B. Overrepresentation of unaccompanied refugee minors in inpatient psychiatric care. *Springerplus* 4: 131. 2015.

38 Daniel-Calveras A, Baldaqui N, Baeza I. Mental health of unaccompanied refugee minors in Europe: A systematic review. *Child Abuse Negl* 2022;133:105865.

39 Armitage AJ, Cohen J, Heys M, Hardelid P, Ward A, Eisen S. Description and evaluation of a pathway for unaccompanied asylum-seeking children. *Arch Dis Child* 2022;107:456-60.

For peer review only

For peer review only - http://bmjopen.bmj.com/site/about/guidelines.xhtml

40 Silverman, M. M., Berman, A. L., Sanddal, N. D., O'carroll, P. W., & Joiner Jr, T. E. (2007b). Rebuilding the tower of Babel: a revised nomenclature for the study of suicide and suicidal behaviors. Part 2: Suicide-related ideations, communications, and behaviors. Suicide and Life-Threatening Behavior, 37(3), 264-277.

41 De Leo, D., Goodfellow, B., Silverman, M., Berman, A., Mann, J., Arensman, E., ... & Kolves, K. (2021). International study of definitions of English-language terms for suicidal behaviours: a survey exploring preferred terminology. BMJ open, 11(2), e043409.

42 Edmondson AJ, Brennan CA, House AO. Non-suicidal reasons for self-harm: A systematic review of self-reported accounts. *J Affect Disord* 2016;191:109-17.

43 Moher D, Shamseer L, Clarke M, *et al*. Preferred reporting items for systematic review and meta-analysis protocols (PRISMA-P) 2015 statement. *Syst Rev* 2015;4:1.

44 World Health Organization. Classification of diseases (ICD), 2018. Available: http://www.who.int/classifications/icd/en/

45 Clarivate Analytics. Endnote X8, 2018. Available: https://endnote. com/ [accessed 8 September 2022].

46 Covidence. Available: https://www.covidence.org/ [accessed 5 Oct 2022].

47 McHugh ML. Interrater reliability: the kappa statistic. *Biochem Med* 2012;22:276-82.

48 Stone JC, Glass K, Clark J, *et al.* A unified framework for bias assessment in clinical research. *Int J Evid Based Healthc* 2019;17:106-20.

49 Borschmann R, Janca E, Carter A, *et al*. The health of adolescents in detention: a global scoping review. *Lancet Public Health* 2020;5:e114-26.

For peer review only - http://bmjopen.bmj.com/site/about/guidelines.xhtml

50 Willoughby M, Young JT, Spittal MJ, *et al*. Violence-related deaths among people released from incarceration: systematic review and meta-analysis of cohort studies. *E Clin Med* 2021;41:101162.

51 Baker WL, Michael White C, Cappelleri JC, *et al*. Understanding heterogeneity in meta-analysis: the role of meta-regression. *Int J Clin Pract* 2009;63:1426-34.

52 Popay J, Roberts H, Sowden A, *et al*. Guidance on the conduct of narrative synthesis in systematic reviews: a product from the ESRC methods program, 2006. Available: https://www.lancaster.ac.uk/media/lancaster-university/content-assets/documents/fhm/dhr/chir/NSsynthesisguidanceVersion1-April2006.pdf

Authors' contributions: KH developed the original research proposal. KH and RB both contributed to the design of the project. KH developed the search strategy, with input from RB. KH wrote the initial draft of the manuscript, and RB contributed significantly to drafting and editing subsequent iterations. Both authors approved the final manuscript.

Funding: This research received no specific grant from any funding agency in the public, commercial, or not-for-profit sectors. RB receives salary and research support from a National Health and Medical Research Council Emerging Leadership Investigator Grant (EL2; GNT2008073).

Competing interests: None declared.

For peer review only

Supplementary appendix 1

Overview of search strategies used for each database

MEDLINE (Ovid) search strategy filtered by date from database inception to 10 February 2023

1. (non-suicidal self-harm or self-injur* or deliberate self-harm or self-poison* or self-mutilat*).af.
2. (migration or immigration or unaccompanied asylum seeker or unaccompanied refugee minor or child or adolescent or teenager).af.
3. 1 and 2

PsycINFO (Ovid) search strategy filtered by date from database inception to 10 February 2023

1. (non-suicidal self-harm or self-injur* or deliberate self-harm or self-poison* or self-mutilat*).af.
2. (migration or immigration or unaccompanied asylum seeker or unaccompanied refugee minor or child or adolescent or teenager).af.
3. 1 and 2

PubMed search strategy filtered by date from database inception to 10 February 2023

(((non-suicidal self-harm or self-injur* or deliberate self-harm or self-poison* or self-mutilat*)) OR ((migration or immigration or unaccompanied asylum seeker or unaccompanied refugee minor or child or adolescent or teenager).af.)) AND (non-suicidal self-harm or self-injur* or deliberate self-harm or self-poison* or self-mutilat* AND migration or immigration or unaccompanied asylum seeker or unaccompanied refugee minor or child or adolescent or teenager)

Scopus search strategy

{non-suicidal self-harm} or {self-injur*} or {deliberate self-harm} or {self-poison*} or {self-mutilat*} OR {migration} or {immigration} or {unaccompanied asylum seeker} or {unaccompanied refugee minor} or child or adolescent or teenager).af.)) AND {non-suicidal self-harm} or {self-injur*} or {deliberate self-harm} or {self-poison*} or {self-mutilat*} AND {migration} or {immigration} or {unaccompanied asylum seeker} or {unaccompanied refugee minor} or child or adolescent or teenager)

For peer review only

PRISMA-P 2015 Checklist

This checklist has been adapted for use with protocol submissions to *Systematic Reviews* from Table 3 in Moher D et al: Preferred reporting items for systematic review and meta-analysis protocols (PRISMA-P) 2015 statement. *Systematic Reviews* 2015 4:1

Section/topic	#	Checklist item	Information reported Yes	Information reported No	Line number(s)
**ADMINISTRATIVE INFORMATION**					
**Title**					
Identification	1a	Identify the report as a protocol of a systematic review	X		p.1
Update	1b	If the protocol is for an update of a previous systematic review, identify as such			N/A
**Registration**	2	If registered, provide the name of the registry (e.g., PROSPERO) and registration number in the Abstract	X		p.3 p.7
**Authors**					
Contact	3a	Provide name, institutional affiliation, and e-mail address of all protocol authors; provide physical mailing address of corresponding author	X		p.1
Contributions	3b	Describe contributions of protocol authors and identify the guarantor of the review	X		p.20
**Amendments**	4	If the protocol represents an amendment of a previously completed or published protocol, identify as such and list changes; otherwise, state plan for documenting important protocol amendments			N/A
**Support**					
Sources	5a	Indicate sources of financial or other support for the review	X		p.20
Sponsor	5b	Provide name for the review funder and/or sponsor			N/A
Role of sponsor/funder	5c	Describe roles of funder(s), sponsor(s), and/or institution(s), if any, in developing the protocol	X		p.20
**INTRODUCTION**					
**Rationale**	6	Describe the rationale for the review in the context of what is already known	X		pp.4-8
**Objectives**	7	Provide an explicit statement of the question(s) the review will address with reference to participants, interventions, comparators, and outcomes (PICO)	X		pp.8-9

BioMed Central
The Open Access Publisher

For peer review only - http://bmjopen.bmj.com/site/about/guidelines.xhtml

Section/topic	#	Checklist item	Information reported: Yes	Information reported: No	Line number(s)
**METHODS**					
**Eligibility criteria**	8	Specify the study characteristics (e.g., PICO, study design, setting, time frame) and report characteristics (e.g., years considered, language, publication status) to be used as criteria for eligibility for the review	X		pp.8-9
**Information sources**	9	Describe all intended information sources (e.g., electronic databases, contact with study authors, trial registers, or other grey literature sources) with planned dates of coverage	X		pp.10-11
**Search strategy**	10	Present draft of search strategy to be used for at least one electronic database, including planned limits, such that it could be repeated	X		p.10
STUDY RECORDS					
Data management	11a	Describe the mechanism(s) that will be used to manage records and data throughout the review	X		pp.10-11
Selection process	11b	State the process that will be used for selecting studies (e.g., two independent reviewers) through each phase of the review (i.e., screening, eligibility, and inclusion in meta-analysis)	X		pp.10-11
Data collection process	11c	Describe planned method of extracting data from reports (e.g., piloting forms, done independently, in duplicate), any processes for obtaining and confirming data from investigators	X		p.10-11
**Data items**	12	List and define all variables for which data will be sought (e.g., PICO items, funding sources), any pre-planned data assumptions and simplifications	X		p. 8 -9 pp.10-11
**Outcomes and prioritization**	13	List and define all outcomes for which data will be sought, including prioritization of main and additional outcomes, with rationale	X		pp.11-12
**Risk of bias in individual studies**	14	Describe anticipated methods for assessing risk of bias of individual studies, including whether this will be done at the outcome or study level, or both; state how this information will be used in data synthesis	X		pp.12-13
DATA					
**Synthesis**	15a	Describe criteria under which study data will be quantitatively synthesized	X		pp.12-13
	15b	If data are appropriate for quantitative synthesis, describe planned summary measures, methods of handling data, and methods of combining data from studies, including any planned exploration of consistency (e.g., I^2, Kendall's tau)	X		pp. 12-13
	15c	Describe any proposed additional analyses (e.g., sensitivity or subgroup analyses, meta-regression)	X		p.12-13
	15d	If quantitative synthesis is not appropriate, describe the type of summary planned	X		p.13
**Meta-bias(es)**	16	Specify any planned assessment of meta-bias(es) (e.g., publication bias across studies, selective	X		p.12-13

3

Section/topic	#	Checklist item	Information reported		Line number(s)
			Yes	No	
		reporting within studies)			
**Confidence in cumulative evidence**	17	Describe how the strength of the body of evidence will be assessed (e.g., GRADE)	X	☐	p.12-13

BioMed Central
The Open Access Publisher

BMJ Open

Self-harm among unaccompanied asylum seekers and refugee minors: protocol for a global systematic review of prevalence, methods, and characteristics

Journal:	*BMJ Open*
Manuscript ID	bmjopen-2022-069237.R2
Article Type:	Protocol
Date Submitted by the Author:	20-Apr-2023
Complete List of Authors:	Hedrick, Kyli; Uppsala Universitet, Department of Public Health and Caring Sciences; The University of Melbourne, Centre for Mental Health, Melbourne School of Population and Global Health<br>Borschmann, Rohan; The University of Melbourne, Centre for Mental Health, Melbourne School of Population and Global Health; Oxford University, Department of Psychiatry, Warneford Hospital
**Primary Subject Heading**:	Mental health
Secondary Subject Heading:	Public health
Keywords:	MENTAL HEALTH, PUBLIC HEALTH, Suicide & self-harm < PSYCHIATRY, Child & adolescent psychiatry < PSYCHIATRY

SCHOLARONE™
Manuscripts

Self-harm among unaccompanied asylum seekers and refugee minors: protocol for a global systematic review of prevalence, methods, and characteristics

Kyli Hedrick[1,2], Rohan Borschmann[2,3,4,5]

[1]Child Health and Parenting (CHAP), Department of Public Health and Caring Sciences, Uppsala University, BMC, Husargatan 3, 753 27, Uppsala, Sweden.

[2]Centre for Mental Health; Melbourne School of Population and Global Health, The University of Melbourne, Melbourne, Victoria, 3010, Australia

[3]Department of Psychiatry; University of Oxford, Warneford Hospital, Oxford OX3 7JX, UK

[4]Centre for Adolescent Health; Murdoch Children's Research Institute, Melbourne, Victoria, 3052, Australia

[5]Melbourne School of Psychological Sciences; University of Melbourne, Melbourne, Victoria, 3010, Australia

[6]School of Population Health, Curtin University, Western Australia, 6845, Australia

Corresponding author: Dr. Kyli Hedrick

Email: kyli.hedrick@pubcare.uu.se

For peer review only - http://bmjopen.bmj.com/site/about/guidelines.xhtml

Abstract

Introduction**:** Asylum seekers and refugees are at an elevated risk of self-harm, with younger age and traumatic experiences found to further increase such risk. Despite this, evidence regarding self-harm among unaccompanied asylum seekers and refugee minors has not been synthesised. As self-harm among minors is a risk factor for a range of adverse clinical and social outcomes, including suicide, such information may help to inform evidence-based prevention strategies among these vulnerable populations. This systematic review will synthesise findings from the literature regarding the prevalence, methods, and characteristics of self-harm, including risk and protective factors, among unaccompanied asylum seekers and refugee minors internationally.

Methods and analysis**:** We searched key electronic databases (PsycINFO, Scopus, PubMed, and Ovid MEDLINE) and grey literature for relevant studies published in English from database inception to 10 February 2023. Our primary outcome is self-harm among unaccompanied asylum seekers and/or refugee minors. With the exception of single case studies, clinical trials, and case-control studies, we will include all types of study design that examine the prevalence of self-harm in unaccompanied asylum seekers and/or refugee minors. We will exclude dissertations, conference abstracts, letters, book chapters, editorials, study registrations, registered protocols, and qualitative studies. Only studies reporting on participants aged <18 years will be eligible for inclusion. The Methodological Standard for Epidemiological Research (MASTER) scale will be used to assess the quality of included studies. If there are sufficient studies, and homogeneity between them, we will conduct meta-analyses to calculate pooled estimates of self-harm rates, as well as comparisons between sub-groups of relevance. If the studies do not report sufficient data, or there is substantial heterogeneity, we will provide a narrative synthesis of the findings.

For peer review only - http://bmjopen.bmj.com/site/about/guidelines.xhtml

Ethics and dissemination: This review is exempt from ethics approval. Our findings will be disseminated through peer-reviewed publications and conference presentations.

PROSPERO registration number CRD42021292709.

Strengths and limitations of this study

- This systematic review uses a comprehensive search strategy including four key academic databases and a grey literature search.
- A strength of this study is that only studies involving unaccompanied asylum seekers and refugee minors and other immigrant populations, and that distinguish between these populations (i.e., consider these populations to be heterogenous), will be included.
- Studies that examine self-harm and suicide attempts but do not distinguish between these acts will be excluded as these two outcomes are qualitatively and motivationally distinct from one another.
- A small number of primary studies, and heterogeneity between study populations and/or study design, may preclude meta-analysis or direct comparison between studies.

For peer review only

For peer review only - http://bmjopen.bmj.com/site/about/guidelines.xhtml

Introduction

Globally, the number of people forcibly displaced because of war, conflict, and political unrest has reached record highs. According to the United Nations High Commissioner for Refugees (UNHCR) [1], as at the end of May 2022, there are now over 100 million forcibly displaced people around the world. Children and young people are markedly over-represented in this humanitarian crisis, accounting for an estimated 41% of those displaced, though they make up 30% of the world's population [2]. Of the 24,100 children who arrived in Greece, Italy, Bulgaria, Spain, Cyprus, and Malta in the 12-month period to December 2021, 17,200 (71%) were unaccompanied and separated [3]. The UNHCR defines a refugee as a person who: 1) has a well-founded fear of being persecuted for reasons relating to race, religion, nationality, membership of a particular social group, or political opinion; 2) is outside the country of (their) nationality; and 3) is unable, or owing to such fear, is unwilling, to avail (themselves) of the protection of that country (p.14) [4]. An asylum seeker is defined as an individual who is seeking refugee 'protection, but whose claim for refugee status has not yet been determined' [5]. Unaccompanied asylum seekers and refugee minors are defined by the United Nations Convention on the Rights of the Child [6] as children or adolescents younger than 18 years who have been separated from both parents and relatives and are not being cared for by an adult, who, by law or custom, is responsible for doing so.

First-time asylum applications from unaccompanied minors in the European Union (EU) Member States with available data increased by 10% between May and June 2022, and 63% between June 2021 and June 2022 [7]. Whilst most unaccompanied minors are males aged between 14-17 years, numbers of female and younger populations are increasing [8]. In 2022, the main countries of origin for unaccompanied minors in the EU Member States were Afghanistan, Syria, Somalia, and Pakistan [7]. In the 2021 financial year, the United States

For peer review only

(US) reported receiving an all-time record of unaccompanied minors from Central America at or near the US-Mexico border, including 145,000 minors [9].

Many individuals who have been forcibly displaced have been subjected to a range of potentially traumatic events in the pre-, peri-, and post-migration phases [10]. These include witnessing the death of a family member or loved one, war, dangerous flight, poor humanitarian conditions, and periods of detention [10, 11], followed by the challenges associated with managing the uncertainty of the asylum process, and the resettlement process [12]. Young people who have been forcibly displaced must navigate such adverse experiences during a challenging developmental period, which may further compound the detrimental impact of these events on their mental health [13]. For young people who are forcibly displaced without parents or caregivers, they must not only navigate several potentially traumatic experiences during a difficult time developmentally, but they must do so without the support and guidance of a parent or caregiver, and whilst managing the loss associated with separation from parents and/or family [14]. Upon arrival in host countries, unaccompanied minors may also be confronted with persistent questioning by authorities about their age, familial relationships, and reason(s) for leaving their home country [15]. In addition, they may be subjected to invasive and imprecise age assessments such as carpal, collarbone, and/or dental x-rays to establish the accuracy of their self-reported age [16, 17]. The particular vulnerabilities and risks faced by unaccompanied asylum seekers and refugee minors have been increasingly acknowledged, paving the way for their entitlement to international protection [6], as well as programs promoting their physical, mental, and educational development [6, 18].

Research has documented the numerous adverse mental health consequences for unaccompanied asylum seekers and refugee minors across several settings and locations [19]. For example, a 2019 global systematic review of the mental health of unaccompanied refugee

For peer review only - http://bmjopen.bmj.com/site/about/guidelines.xhtml

minors by von Wethern and colleagues [19] found that despite varying estimates of psychological difficulties, rates of PTSD (17-85%), depression (13-76%), and anxiety (11-85%) were significantly higher in unaccompanied refugee minors than those found in the general western population. Evidence compiled by von Werthern et al. [19] also found that adolescence and being female were indicators of increased risk of psychiatric disorders. In addition, the authors' review highlighted that there was significantly more exposure to traumatic events such as physical violence, torture, imprisonment, and killings among unaccompanied minors, compared with exposure in the general population [20, 21, 22, 23]. Earlier systematic reviews have largely focused on the mental health and wellbeing of unaccompanied minors [19, 24, 25, 26], or the mental health of refugee minors [27]. However, preliminary searches conducted by the authors for the purposes of this study (in PROSPERO, the PUBMED/MEDLINE and DARE databases, and the JBI Evidence Synthesis journal [28]) indicate that no reviews have examined self-harm (elsewhere also described as non-suicidal self-injury [NSSI], and defined as the deliberate, self-inflicted destruction of body tissue, without suicidal intent, and for purposes not socially or culturally sanctioned [29]) in unaccompanied asylum seekers and refugee minors globally. As self-harm is common among young people [30, 31], and this risk is further elevated among those with experiences of trauma [32], it is conceivable that unaccompanied asylum seekers and refugee minors are at even greater risk of self-harm due to their younger age and prior experience of a range of potentially traumatic events in the pre-, peri-, and post-migration phases [10]. Should a higher prevalence of self-harm among unaccompanied asylum seekers and refugee minors be identified, these findings would elucidate the need for providing mental health support at the earliest available opportunity for such minors. Indeed, given that self-harm is a risk factor for severe mental illness, as well as for all-cause mortality [33], self-harm is an outcome of marked importance. Furthermore, as every act of self-harm (including explicitly

For peer review only - http://bmjopen.bmj.com/site/about/guidelines.xhtml

non-suicidal actions and behaviour) has the potential to be lethal as a result of accident or misadventure, and self-harm is the strongest risk factor for suicide [33, 34], self-harm (irrespective of intent) is an extremely important outcome to target. Furthermore, as the social and public health costs of self-harm are known to be high [34, 35], the synthesis of such evidence may help to inform distribution of scarce public resources. One broader review that focused on self-harming behaviours among immigrants in Europe by Garguilo and colleagues [36] did report – via a synthesis of three pertinent studies - that unaccompanied minors were an 'at-risk' group for self-harm in the European context. However, as unaccompanied minors were not explicitly included in Garguilo and colleagues' [36] search strategy, and the setting of their review was European, rather than global, it is likely that other salient studies may have been excluded. As such, a comprehensive global review synthesising the evidence relating to self-harm among unaccompanied asylum seekers and refugee minors could inform evidence-based prevention and management strategies in these vulnerable populations.

A Swedish study by Ramel and colleagues [37] was included in both von Werthern and colleagues [19] and Garguilo and colleagues [36] reviews. In this study, the authors compared the psychiatric care of unaccompanied refugee minors and accompanied refugee minors (with an average age of 15.1 years), reporting that 3.4% (or 56/1,657) of unaccompanied refugee minors received inpatient psychiatric care and 0.67% involuntary care, compared to 0.26% (205/81,077) and 0.02% respectively of the accompanied refugee minors [37]. Both comparisons were statistically significant ($p < 0.001$). In addition, Ramel and colleagues found that a higher proportion of unaccompanied refugee minors (21.8; 76%) than accompanied minors (21.1; 56%) exhibited self-harm or suicidal behaviour on admission to hospital ($p < 0.001$) [37]. The study also noted that 86% (13.2) of unaccompanied minors were hospitalised due to the stress associated with the asylum process [37]. A 2022 systematic review of the mental health of unaccompanied minors in Europe by Daniel-

For peer review only - http://bmjopen.bmj.com/site/about/guidelines.xhtml

Calveras and colleagues [38] also included one retrospective study evaluating data from 101 unaccompanied refugee minors (aged 14-17) presenting to health care providers in the United Kingdom, reporting that 8% (8/101) had engaged in self-harm or suicidal behaviour [39]. None of the studies included in the reviews by von Werthern [19], Garguilo [36], or Daniel-Calveras and colleagues [38] (or the original authors of the two studies highlighted above [37, 39]), however, separated out or distinguished – where possible – episodes of self-harm from suicidal behaviour when presenting their findings, though Garguilo and colleagues [36] did state in their discussion that self-harm should be viewed as a distinct clinical entity. Indeed, definitions of self-harming behaviour have varied historically [40, 41], and behavioural intent is difficult to measure. Despite this, as self-harm and suicide attempts are motivationally and qualitatively distinct from one another [42], differentiating between the two – where possible – is critical to inform the provision of appropriate physical and mental health care. Furthermore, as no terms relating to self-harm were included in von Werthern and colleagues' [19] or Daniel-Calveras and colleagues' [38] search strategies, and those included in Garguilo et al.'s [36] search strategy were not exhaustive, additional studies focusing specifically on self-harm (as distinct from suicidal behaviour) may not have been identified in these reviews.

We aim to synthesise the evidence regarding the prevalence, methods, and characteristics of self-harm, including risk and protective factors, among unaccompanied asylum seekers and refugee minors internationally.

Methods and analysis

This protocol is reported in accordance with the Preferred Reporting Items for Systematic Reviews and Meta-Analysis Protocols (PRISMA-P) [43].

For peer review only - http://bmjopen.bmj.com/site/about/guidelines.xhtml

Patient and Public Involvement

There was no patient or public involvement in the design of this study.

Eligibility criteria

Participants

We will include studies examining self-harm among unaccompanied asylum seekers and refugee minors – defined as a person aged <18 years who has been separated from both parents and relatives and is not being cared for by an adult, who, by law or custom, is responsible for doing so [6] – in any country. Studies reporting on unaccompanied asylum seekers and/or refugee minors and other immigrant populations, but that do not distinguish between these populations, will be excluded. Studies reporting on suicide or suicide attempts only will be excluded. Where studies refer to suicide and self-harm separately, only findings regarding self-harm will be included. Comparison studies involving accompanied asylum seekers and/or refugee minors, as well and the general/native population in countries of origin, transit, and/or resettlement will also be included, where available.

Outcome measure

Our primary outcome measure will be self-harm. For the purposes of this review, self-harm, elsewhere also described as non-suicidal self-injury [NSSI], is defined as the deliberate, self-inflicted destruction of body tissue, without suicidal intent, and for purposes not socially or culturally sanctioned [29]. It may include behaviours such as cutting, burning, biting, and scratching skin [29]. Self-harm may be measured by self-report, clinical interview, or administrative data (e.g., emergency department [ED], hospital, or incident reports). An additional outcome measure will be method(s) used to self-harm. Methods of self-harm may also be measured by self-report, clinical interview, or administrative data, including the

For peer review only - http://bmjopen.bmj.com/site/about/guidelines.xhtml

World Health Organization's International Classification of Diseases Tenth Revision (ICD-10) codes [44].

Study design

We will include published cohort studies (prospective and retrospective) of unaccompanied asylum seekers and refugee minors which report on the prevalence of self-harm. We will also include grey literature (see "Information sources and search strategy" below). We will exclude single case studies, dissertations, conference abstracts, letters, book chapters, editorials, study registrations, clinical trials, case-control studies, registered protocols, and qualitative studies. We will not include previous systematic reviews, as not all included studies may not meet our inclusion criteria. We will, however, identify any peer-reviewed studies related to any dissertations, conference abstracts, study registrations, and studies assessed for previous reviews that meet our inclusion criteria but were not identified via our search strategy. Only studies published in English will be included.

Information sources and search strategy

We searched four key health and medical databases (PsycINFO, Scopus, PubMed, and OVID MEDLINE) for relevant literature published in English using key terms relating to self-harm, migration, and minors, from database inception until the date of our final search (10 February 2023). The MEDLINE search strategy is outlined in table 1. The full search strategy for each database is outlined in Online supplemental appendix 1.The electronic database searches will be supplemented by reviewing the reference lists of eligible articles, as well as searching the websites of relevant non-governmental organisations who have worked with unaccompanied asylum seekers and refugee minors (such as Médecins sans Frontières and Save the Children), for pertinent grey literature.

For peer review only - http://bmjopen.bmj.com/site/about/guidelines.xhtml

Table 1

MEDLINE search strategy

1.	(non-suicidal self-harm or self-injur* or deliberate self-harm or self-poison* or self-mutilat*).af.
2.	(migration or immigration or unaccompanied asylum seeker or unaccompanied refugee minor or child or adolescent or teenager).af.
3.	1 and 2

Study selection

All studies identified through the database search will be downloaded to Endnote [45] and duplicates removed. The remaining studies will be imported into Covidence [46] for screening. All titles and abstracts will be screened for inclusion by the primary author (KH), with a random 20% also screened by the second author (RB). After 20% of the papers identified in the search strategy have been double screened, we will reassess our eligibility criteria to ensure that they are relevant to the studies that are identified. The reassessment process will involve a discussion between the reviewers, with any differences or uncertainty regarding study inclusion resolved by a third researcher. The overall inter-rater reliability for the title and abstract screening will be calculated using Cohen's kappa statistic [47]. If reliability is low (<0.40),[47] the authors will review the eligibility criteria, double-code a second random 10% of retrieved studies and recalculate Cohen's kappa statistic [47]. Studies will be coded as either 0=Does not meet eligibility criteria, or 1=Meets eligibility criteria or the full-text article needs to be screened to confirm eligibility. After title and abstract screening is complete, all remaining full-text articles will be independently screened in duplicate by KH and RB, with any conflicts related to study inclusion resolved through discussion with a third researcher. Where clarification is needed to determine eligibility, we will make a maximum of three attempts to contact the original study author(s), with no more attempts at contact made after two months.

For peer review only

Data extraction

Data extraction will be conducted by KH using a standardised extraction form developed by the researchers and will be checked by a second reviewer (RB). The following data will be extracted from each study: author(s), study year, country of study, study design, setting, sample size, sample characteristics (e.g., gender, age, country of origin, country/ies of transit and/or resettlement, and length of separation from parents/caregivers, where possible), reported prevalence of self-harm, method(s) used to self-harm, risk and protective factors associated with self-harm, outcomes measure(s) used, characteristics of findings including incidence or episode rates, 95% CIs, p-values and effect sizes (where reported). A maximum of three attempts will be made to contact study authors for further information if any of the required data are missing, incomplete, or unclear.

Risk of bias

The Methodological Standard for Epidemiological Research (MASTER) [48] scale will be used to assess the quality of included studies. The quality and risk of bias will be independently appraised in duplicate by KH and RB. The two reviewers will then compare scores with any uncertainty resolved through discussion and consensus before allocation of final appraisal scores. The MASTER scale [48] provides a single consolidated tool to assess the risk of bias across different types of study design. This is done by assessing each study for the presence of several methodological standards aimed at addressing the risk of bias across six potential bias domains (selection, information, design-related, analytic, confounding, and external-validity) [48]. Using the approach taken in previous systematic reviews [49, 50], we will discuss the possible risk of bias and study quality in text, as well as generate a score summarising each study's quality by using the proportion of safeguards against bias that each study incorporated.

For peer review only - http://bmjopen.bmj.com/site/about/guidelines.xhtml

Data synthesis

We will provide a descriptive overview of the included studies, including the study year(s), design, size, and location of the study sample, measure(s) used to report self-harm, and any associated characteristics.

If a sufficient number of studies report on the rates of self-harm, or associated characteristics of self-harm, and there is sufficient homogeneity between studies (by, for example, design, sample, or outcome), we will conduct meta-analyses to calculate pooled estimates of self-harm rates. Heterogeneity will be assessed using the I^2 statistic.

If the number of included studies is sufficient, we will use meta-regression [51] to investigate the influence of gender, age, country of origin, and length of separation from parents/caregivers on rates of self-harm. To investigate the impact of study quality on risk of bias, we will conduct a sensitivity analysis which includes only papers rated as high-quality (i.e., those papers assessed as scoring above the median in the MASTER [48] scale).

If meta-analyses are not possible due to insufficient data on the rates or associated characteristics of self-harm, or if there is substantial heterogeneity, a narrative synthesis will be created (as per established guidelines on the conduct of a narrative synthesis [52]). This descriptive overview will provide information in both text and table format to summarise and explain the included study findings, based on outcome, associated characteristics of self-harm, and geographic region. From this, we may also develop a theory [52] of self-harm among unaccompanied asylum seekers and refugee minors, and the factors that influence it, and an assessment of the overall robustness of this summary.

Ethics and dissemination

As this is a review of studies that have already obtained ethics approval, this study is exempt from ethics approval. The findings of our review will be disseminated in a peer-reviewed journal article and via presentations at relevant national and international conferences.

For peer review only - http://bmjopen.bmj.com/site/about/guidelines.xhtml

Ethics statements

Patient consent for publication

Not applicable.

For peer review only

For peer review only - http://bmjopen.bmj.com/site/about/guidelines.xhtml

References

1 United Nations High Commissioner for Refugees. A record 100 million people forcibly displaced worldwide, 2022. Available: https://news.un.org/en/story/2022/05/1118772

2 United Nations High Commissioner for Refugees. Global trends report, 2021. Available: https://www.unhcr.org/62a9d1494/global-trends-report-2021

3 UNICEF. Refugee and migrant children in Europe, January – December, 2021. Available: https://www.unicef.org/eca/media/23466/file/Refugee%20and%20Migrant%20Children%20in%20Europe%20.pdf

4 United Nations High Commissioner for Refugees. Convention and Protocol Relating to the Status of Refugees, 2010. Available: http://www.unhcr.org/3b66c2aa10.html

5 Phillip J. Asylum seekers and refugees. What are the facts? 2015. Available: https://www.aph.gov.au/About_Parliament/Parliamentary_Departments/Parliamentary_Library/pubs/rp/rp1415/AsylumFacts

6 UN General Assembly, Convention on the Rights of the Child, 20 November 1989, United Nations, Treaty Series, vol. 1577, p. 3. Available: https://www.refworld.org/docid/3ae6b38f0.html

7 Eurostat. First time asylum applicants up 4% in June 2022. Available: https://ec.europa.eu/eurostat/web/products-eurostat-news/-/ddn-20220926

8 Eurostat. Unaccompanied minor asylum applicants by type of applicant, citizenship, age and sex - monthly data (rounded) 30 September, 2022. Available:

For peer review only - http://bmjopen.bmj.com/site/about/guidelines.xhtml

https://ec.europa.eu/eurostat/databrowser/view/MIGR_ASYUMACTM/default/table?lang=en&category=migr.migr_asy.migr_asyapp.migr_asyum

9 U.S. Customs and Border Protection. Southwest land border encounters 2021. Available: https://www.cbp.gov/newsroom/stats/southwest-land-border-encounters

10 Priebe S, Giacco D, El-Nagib R. Public health aspects of mental health among migrants and refugees: a review of the evidence on mental health care for refugees, asylum seekers and irregular migrants in the WHO European Region. Copenhagen: WHO Regional Office for Europe; 2016. (Health Evidence Network [HEN] Synthesis Report 47). Available: https://www.ncbi.nlm.nih.gov/books/NBK391045/

11 Porter M, Haslam N. Predisplacement and postdisplacement factors associated with mental health of refugees and internally displaced persons: a meta-analysis. *JAMA* 2005;294:602-12.

12 Li SSY, Liddell BJ, Nickerson A. The relationship between post-migration stress and psychological disorders in refugees and asylum seekers. *Curr Psychiatry Rep* 2016;18:82.

13 Hodes M, Vostanis P. Practitioner Review: Mental health problems of refugee children and adolescents and their management. *J Child Psychol Psychiatry* 2019;60:716-31.

14 Vervliet M, Meyer Demott MA, Jakobsen M, Broekaert E, Heir T, Derluyn I. The mental health of unaccompanied refugee minors on arrival in the host country. *Scand J Psychol* 2014;55:33-7.

15 Bean T, Derluyn I, Eurelings-Bontekoe E, Broekaert E, Spinhoven P. Comparing psychological distress, traumatic stress reactions, and experiences of unaccompanied

For peer review only - http://bmjopen.bmj.com/site/about/guidelines.xhtml

refugee minors with experiences of adolescents accompanied by parents. *J Nerv Ment Dis* 2007;195:288-97.

16 Noll G. Junk Science? Four arguments against the radiological age assessment of unaccompanied minors seeking asylum. *Int J Ref Law* 2016;28:234-50.

17 Aynsley-Green A, Cole TJ, Crawley H, Lessof N, Boag LR, Wallace RMM. Medical, statistical, ethical and human rights considerations in the assessment of age in children and young people subject to immigration control. *Br Med Bull* 2012;102:17-42.

18 European Commission, Directorate-General for Justice and Consumers. EU strategy on the rights of the child, 2021. Available: https://data.europa.eu/doi/10.2838/313794

19 von Werthern M, Grigorakis G, Vizard E. The mental health and wellbeing of Unaccompanied Refugee Minors (URMs). *Child Abuse Negl* 2019;98:104146.

20 Derluyn I, Mels C, Broekaert E. Mental health problems in separated refugee adolescents. *J Adolesc Health* 2009;44:291-7.

21 Kirk Felsman J, Leong FTL, Johnson MC, Crabtree Felsman I. Estimates of psychological distress among Vietnamese refugees: Adolescents, unaccompanied minors and young adults. *Soc. Sci. Med* 1990;3:1251-6.

22 Hodes M, Jagdev D, Chandra N, Cunniff A. Risk and resilience for psychological distress amongst unaccompanied asylum seeking adolescents. *J Child Psychol Psychiatry* 2008;49:723-32.

23 Pinto Wiese EB, Burhorst I. The mental health of asylum-seeking and refugee children and adolescents attending a clinic in the Netherlands. *Transcult Psychiatry* 2007;44:596-613.

For peer review only - http://bmjopen.bmj.com/site/about/guidelines.xhtml

24 Bamford J, Fletcher M, Leavey G. Mental health outcomes of unaccompanied refugee minors: a rapid review of recent research. *Curr Psychiatry Rep* 2021;23(8):46.

25 Höhne E, van der Meer AS, Kamp-Becker I, Christiansen H. A systematic review of risk and protective factors of mental health in unaccompanied minor refugees. *Eur Child Adolesc Psychiatry* 2022;3:1-15.

26 Huemer J, Karnik NS, Voelkl-Kernstock S, Granditsch E, Dervic K, Friedrich MH, et al. Mental health issues in unaccompanied refugee minors. *Child Adolesc Psychiatry Ment Health* 2009;3:13.

27 Fazel M, Reed RV, Panter-Brick C, Stein A. Mental health of displaced and refugee children resettled in high-income countries: risk and protective factors. *Lancet* 2012;379:266-82.

28 Munn Z, Moola S, Lisy K, Riitano D, Tufanaru C. Chapter 5: Systematic reviews of prevalence and incidence. In: Aromataris E, Munn Z (Editors). *JBI Manual for Evidence Synthesis.* JBI, 2020. Available: https://synthesismanual.jbi.global. https://doi.org/10.46658/JBIMES-20-06

29 International Society for the Study of Self-Injury. Definition of non-suicidal self-injury, 2014. Available: https://www.itriples.org/what-is-nssi.

30 Moran, P., Coffey, C., Romaniuk, H., Olsson, C., Borschmann, R., Carlin, J.B., & Patton, G.C. (2011). The natural history of self-harm during adolescence and young adulthood: population-based cohort study. *The Lancet*, *379(9812)*, 236-243.

31 Borschmann, R, Mundy, LK, Canterford, L, Moreno-Betancur, M, Moran, PA, Viner, RM, Allen, NB, Degenhardt, L, Kosoloa, S, Fedyzsyn, I, Patton, GC. (2020). Self-

For peer review only - http://bmjopen.bmj.com/site/about/guidelines.xhtml

harm in primary school-aged children: prospective cohort study. *PLOS ONE* (in press).

32 Fazel S, Runeson B. Suicide. *N Engl J Med* 2020;382:266-74.

33 Hawton K, Bale L, Brand F, et al. Mortality in children and adolescents following presentation to hospital after non-fatal self-harm in the Multicentre Study of Self-harm: a prospective observational cohort study. *Lancet Child Adolesc Health*. 2020;4(2):111-120. doi:10.1016/S2352-4642(19)30373

34 Mehlum L. Cost of self-harm to society is high and increasing: a call for evidence-based and systematic treatment approaches. *Acta Psychiatrica Scandinavica*. 2022;145(4):317-8.

35 Mitchell RJ, Seah R, Ting HP, Curtis K, Foster K. Intentional self-harm and assault hospitalisations and treatment cost of children in Australia over a 10-year period. *Australian and New Zealand Journal of Public Health*. 2018;42(3):240-6.

36 Gargiulo A, Tessitore F, Le Grottaglie F, Margherita G. Self-harming behaviours of asylum seekers and refugees in Europe: A systematic review. *Int J Psychol* 2021;56:189-98.

37 Ramel B, Täljemark J, Lindgren A, Johansson B. Overrepresentation of unaccompanied refugee minors in inpatient psychiatric care. *Springerplus* 4: 131. 2015.

38 Daniel-Calveras A, Baldaqui N, Baeza I. Mental health of unaccompanied refugee minors in Europe: A systematic review. *Child Abuse Negl* 2022;133:105865.

For peer review only - http://bmjopen.bmj.com/site/about/guidelines.xhtml

39 Armitage AJ, Cohen J, Heys M, Hardelid P, Ward A, Eisen S. Description and evaluation of a pathway for unaccompanied asylum-seeking children. *Arch Dis Child* 2022;107:456-60.

40 Silverman, M. M., Berman, A. L., Sanddal, N. D., O'carroll, P. W., & Joiner Jr, T. E. (2007b). Rebuilding the tower of Babel: a revised nomenclature for the study of suicide and suicidal behaviors. Part 2: Suicide-related ideations, communications, and behaviors. Suicide and Life-Threatening Behavior, 37(3), 264-277.

41 De Leo, D., Goodfellow, B., Silverman, M., Berman, A., Mann, J., Arensman, E., ... & Kolves, K. (2021). International study of definitions of English-language terms for suicidal behaviours: a survey exploring preferred terminology. BMJ open, 11(2), e043409.

42 Edmondson AJ, Brennan CA, House AO. Non-suicidal reasons for self-harm: A systematic review of self-reported accounts. *J Affect Disord* 2016;191:109-17.

43 Moher D, Shamseer L, Clarke M, *et al*. Preferred reporting items for systematic review and meta-analysis protocols (PRISMA-P) 2015 statement. *Syst Rev* 2015;4:1.

44 World Health Organization. Classification of diseases (ICD), 2018. Available: http://www.who.int/classifications/icd/en/

45 Clarivate Analytics. Endnote X8, 2018. Available: https://endnote. com/ [accessed 8 September 2022].

46 Covidence. Available: https://www.covidence.org/ [accessed 5 Oct 2022].

47 McHugh ML. Interrater reliability: the kappa statistic. *Biochem Med* 2012;22:276-82.

48 Stone JC, Glass K, Clark J, *et al.* A unified framework for bias assessment in clinical research. *Int J Evid Based Healthc* 2019;17:106-20.

For peer review only

For peer review only - http://bmjopen.bmj.com/site/about/guidelines.xhtml

49 Borschmann R, Janca E, Carter A, *et al*. The health of adolescents in detention: a global scoping review. *Lancet Public Health* 2020;5:e114-26.

50 Willoughby M, Young JT, Spittal MJ, *et al*. Violence-related deaths among people released from incarceration: systematic review and meta-analysis of cohort studies. *E Clin Med* 2021;41:101162.

51 Baker WL, Michael White C, Cappelleri JC, *et al*. Understanding heterogeneity in meta-analysis: the role of meta-regression. *Int J Clin Pract* 2009;63:1426-34.

52 Popay J, Roberts H, Sowden A, *et al*. Guidance on the conduct of narrative synthesis in systematic reviews: a product from the ESRC methods program, 2006. Available: https://www.lancaster.ac.uk/media/lancaster-university/content-assets/documents/fhm/dhr/chir/NSsynthesisguidanceVersion1-April2006.pdf

Authors' contributions: KH developed the original research proposal. KH and RB both contributed to the design of the project. KH developed the search strategy, with input from RB. KH wrote the initial draft of the manuscript, and RB contributed significantly to drafting and editing subsequent iterations. Both authors approved the final manuscript.

Funding: This research received no specific grant from any funding agency in the public, commercial, or not-for-profit sectors. RB receives salary and research support from a National Health and Medical Research Council Emerging Leadership Investigator Grant (EL2; GNT2008073).

Competing interests: None declared.

For peer review only

Supplementary appendix 1

Overview of search strategies used for each database

MEDLINE (Ovid) search strategy filtered by date from database inception to 10 February 2023

1. (non-suicidal self-harm or self-injur* or deliberate self-harm or self-poison* or self-mutilat*).af.
2. (migration or immigration or unaccompanied asylum seeker or unaccompanied refugee minor or child or adolescent or teenager).af.
3. 1 and 2

PsycINFO (Ovid) search strategy filtered by date from database inception to 10 February 2023

1. (non-suicidal self-harm or self-injur* or deliberate self-harm or self-poison* or self-mutilat*).af.
2. (migration or immigration or unaccompanied asylum seeker or unaccompanied refugee minor or child or adolescent or teenager).af.
3. 1 and 2

PubMed search strategy filtered by date from database inception to 10 February 2023

(((non-suicidal self-harm or self-injur* or deliberate self-harm or self-poison* or self-mutilat*)) OR ((migration or immigration or unaccompanied asylum seeker or unaccompanied refugee minor or child or adolescent or teenager).af.)) AND (non-suicidal self-harm or self-injur* or deliberate self-harm or self-poison* or self-mutilat* AND migration or immigration or unaccompanied asylum seeker or unaccompanied refugee minor or child or adolescent or teenager)

Scopus search strategy

{non-suicidal self-harm} or {self-injur*} or {deliberate self-harm} or {self-poison*} or {self-mutilat*} OR {migration} or {immigration} or {unaccompanied asylum seeker} or {unaccompanied refugee minor} or child or adolescent or teenager).af.)) AND {non-suicidal self-harm} or {self-injur*} or {deliberate self-harm} or {self-poison*} or {self-mutilat*} AND {migration} or {immigration} or {unaccompanied asylum seeker} or {unaccompanied refugee minor} or child or adolescent or teenager)

For peer review only

PRISMA-P 2015 Checklist

This checklist has been adapted for use with protocol submissions to *Systematic Reviews* from Table 3 in Moher D et al: Preferred reporting items for systematic review and meta-analysis protocols (PRISMA-P) 2015 statement. *Systematic Reviews* 2015 4:1

Section/topic	#	Checklist item	Information reported Yes	Information reported No	Line number(s)
**ADMINISTRATIVE INFORMATION**					
**Title**					
Identification	1a	Identify the report as a protocol of a systematic review	X		p.1
Update	1b	If the protocol is for an update of a previous systematic review, identify as such			N/A
**Registration**	2	If registered, provide the name of the registry (e.g., PROSPERO) and registration number in the Abstract	X		p.3 p.7
**Authors**					
Contact	3a	Provide name, institutional affiliation, and e-mail address of all protocol authors; provide physical mailing address of corresponding author	X		p.1
Contributions	3b	Describe contributions of protocol authors and identify the guarantor of the review	X		p.20
**Amendments**	4	If the protocol represents an amendment of a previously completed or published protocol, identify as such and list changes; otherwise, state plan for documenting important protocol amendments			N/A
**Support**					
Sources	5a	Indicate sources of financial or other support for the review	X		p.20
Sponsor	5b	Provide name for the review funder and/or sponsor			N/A
Role of sponsor/funder	5c	Describe roles of funder(s), sponsor(s), and/or institution(s), if any, in developing the protocol	X		p.20
**INTRODUCTION**					
**Rationale**	6	Describe the rationale for the review in the context of what is already known	X		pp.4-8
**Objectives**	7	Provide an explicit statement of the question(s) the review will address with reference to participants, interventions, comparators, and outcomes (PICO)	X		pp.8-9

BioMed Central The Open Access Publisher

Section/topic	#	Checklist item	Information reported		Line number(s)
			Yes	No	
**METHODS**					
**Eligibility criteria**	8	Specify the study characteristics (e.g., PICO, study design, setting, time frame) and report characteristics (e.g., years considered, language, publication status) to be used as criteria for eligibility for the review	☒	☐	pp.8-9
**Information sources**	9	Describe all intended information sources (e.g., electronic databases, contact with study authors, trial registers, or other grey literature sources) with planned dates of coverage	☒	☐	pp.10-11
**Search strategy**	10	Present draft of search strategy to be used for at least one electronic database, including planned limits, such that it could be repeated	☒	☐	p.10
STUDY RECORDS					
Data management	11a	Describe the mechanism(s) that will be used to manage records and data throughout the review	☒	☐	pp.10-11
Selection process	11b	State the process that will be used for selecting studies (e.g., two independent reviewers) through each phase of the review (i.e., screening, eligibility, and inclusion in meta-analysis)	☒	☐	pp.10-11
Data collection process	11c	Describe planned method of extracting data from reports (e.g., piloting forms, done independently, in duplicate), any processes for obtaining and confirming data from investigators	☒	☐	p.10-11
**Data items**	12	List and define all variables for which data will be sought (e.g., PICO items, funding sources), any pre-planned data assumptions and simplifications	☒	☐	p. 8 -9 pp.10-11
**Outcomes and prioritization**	13	List and define all outcomes for which data will be sought, including prioritization of main and additional outcomes, with rationale	☒	☐	pp.11-12
**Risk of bias in individual studies**	14	Describe anticipated methods for assessing risk of bias of individual studies, including whether this will be done at the outcome or study level, or both; state how this information will be used in data synthesis	☒	☐	pp.12-13
DATA					
**Synthesis**	15a	Describe criteria under which study data will be quantitatively synthesized	☒	☐	pp.12-13
	15b	If data are appropriate for quantitative synthesis, describe planned summary measures, methods of handling data, and methods of combining data from studies, including any planned exploration of consistency (e.g., I^2, Kendall's tau)	☒	☐	pp. 12-13
	15c	Describe any proposed additional analyses (e.g., sensitivity or subgroup analyses, meta-regression)	☒	☐	p.12-13
	15d	If quantitative synthesis is not appropriate, describe the type of summary planned	☒	☐	p.13
**Meta-bias(es)**	16	Specify any planned assessment of meta-bias(es) (e.g., publication bias across studies, selective	☒	☐	p.12-13

BioMed Central
The Open Access Publisher

Section/topic	#	Checklist item	Information reported		Line number(s)
			Yes	No	
		reporting within studies)			
**Confidence in cumulative evidence**	17	Describe how the strength of the body of evidence will be assessed (e.g., GRADE)	☒	☐	p.12-13

BioMed Central
The Open Access Publisher
